# Oxygenation alleviates waterlogging-caused damages to cherry rootstocks

**DOI:** 10.1186/s43897-023-00056-1

**Published:** 2023-04-17

**Authors:** Yuxuan Wang, Yan Xu, Jieming Xu, Wanxia Sun, Zhengxin Lv, Muhammad Aamir Manzoor, Xunju Liu, Zhiyu Shen, Jiyuan Wang, Ruie Liu, Matthew D. Whiting, Songtao Jiu, Caixi Zhang

**Affiliations:** 1 Department of Plant Science, School of Agriculture and Biology, Shanghai Jiao Tong University, Shanghai, 200240 China; 2 Department of Horticulture, Washington State University, Prosser, WA 99350 USA

**Keywords:** Cherry, Waterlogging, Hypoxia, Oxygenation, Transcriptome analysis

## Abstract

Waterlogging has occurred more frequently in recent years due to climate change, so it is a huge threat to crop yield and quality. Sweet cherry, a fruit tree with a high economic value, is sensitive to waterlogging stress. One of the most effective methods for enhancing the waterlogging tolerance of sweet cherries is to select waterlogging-tolerant rootstocks. However, the waterlogging tolerance of different cherry rootstocks, and the underlying mechanism remains uncharacterized. Thus, we first evaluated the waterlogging resistance of five sweet cherry rootstocks planted in China. The data showed that ‘Gisela 12’ and ‘Colt’ were the most waterlogging-sensitive and -tolerant among the five tested varieties, respectively. Oxygenation effectively alleviated the adverse impacts of waterlogging stress on cherry rootstocks. Moreover, we found that the waterlogging group had lower relative water content, Fv/Fm value, net photosynthetic rate, and higher antioxidant enzyme activities, whereas the oxygenated group performed better in all these parameters. RNA-Seq analysis revealed that numerous DEGs were involved in energy production, antioxidant metabolism, hormone metabolism pathways, and stress-related transcription factors. These findings will help provide management strategies to enhance the waterlogging tolerance of cherry rootstocks and thereby achieve higher yield and better quality of cherries.

**Graphical Abstract:**

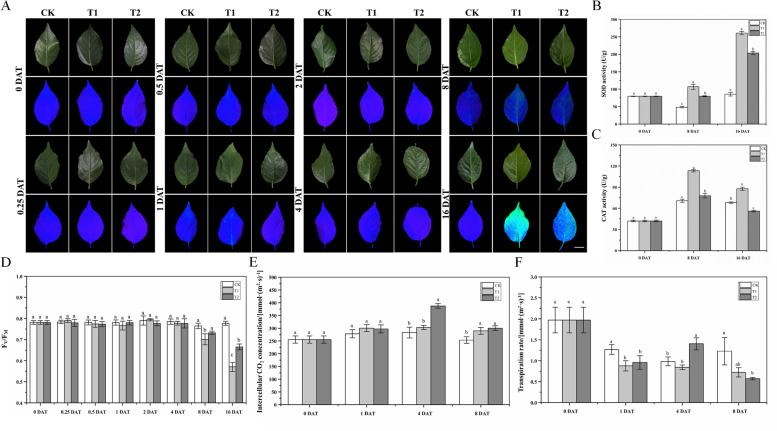

**Supplementary Information:**

The online version contains supplementary material available at 10.1186/s43897-023-00056-1.

## Core

‘Gisela 12’ and ‘Colt’ were the most waterlogging-sensitive and -tolerant among the five tested cherry rootstock varieties, respectively. Oxygenation effectively alleviated the adverse impacts of waterlogging stress on cherry rootstocks. Waterlogging-induced differentially expressed genes (DEGs) were involved in energy production, antioxidant metabolism, hormone metabolism pathways, and in encoding stress-related transcription factors. These findings will help provide some management strategies to enhance the waterlogging tolerance of cherry rootstocks and thereby achieve higher yield and better quality of sweet cherries.

## Gene & accession numbers

The raw data were uploaded to the NCBI Sequence Read Archive (SRA) database (accession number PRJNA866188). A list of genes and sequences used in the qRT-PCR analysis can be found in Supplementary Table S1.

## Introduction

Climatic changes, such as frequent droughts, floods, and extreme temperature events, are expected to be the main drivers of agricultural production costs in the coming years and could cumulatively contribute to terrible economic situations, consequent poverty, and undernourishment (FAO et al., 2020). Among these natural events, floods triggered by climate change have occurred globally at a higher frequency and negatively impact the yield and quality of crops (Hirabayashi et al. 2013; Tanoue et al. 2016). To cope with this crisis, it is crucial to investigate plant flooding tolerance and elucidate the mechanisms underlying crop adaptation to low-oxygen conditions. There are two types of flooding, depending on water depth: waterlogging, in which only roots are flooded, and submergence, in which the whole plant is partially or completely submerged (Bailey-Serres et al. 2012). An oxygen deficit is one of the immediate consequences of waterlogging because the diffusion of oxygen in water is approximately 10,000 times slower than that in air, and the concentration of oxygen infiltrating soil flooded with water is approximately 320,000 times lower than that in gas-filled soil (Voesenek and Bailey-Serres 2015). Because low-oxygen conditions inhibit respiration in roots, plants fulfill their energy needs through anaerobic respiration. However, toxic substances can be produced in anaerobic respiration pathways, i.e., glycolysis and ethanol fermentation, under protracted waterlogging, which can cause cell death and lead to plant senescence, severely affecting photosynthesis (Zhang et al. 2017; Yan et al., 2018). The overground parts of plants were also damaged during waterlogging. Closure of leaf stomata, chlorophyll degradation, yellowing and peeling of leaves occurred under waterlogging led to a decline in plant photosynthetic capacity, which is a crucial factor determining plant lifespan (Kuai et al. 2014; Yan et al., 2018). Chemical measures have been attempted to enhance waterlogging tolerance of plants. Application of exogenous regulatory substances, such as γ-aminobutyric acid and H_2_O_2_, can help plants resist waterlogging (Andrade et al. 2018; Salah et al. 2019). However, studies on how physical methods, such as oxygenation, affect plants under waterlogging conditions are scarce. Moreover, waterlogging tolerance has mostly been studied in model plants and major crops, but rarely in fruit trees.

Sweet cherry (*Prunus avium* L.) is an important woody deciduous fruit tree with high economic value because of its flavor and quality (Martínez-Romero et al. 2006). Given that rootstocks have crucial effects on the stress responses of cherry plants, selecting a rootstock appropriate for the local environment is important. Adaptation of cherry rootstocks to drought and salinity has been reported (Erturk et al. 2007; Sivritepe et al. 2008; Wheeler et al. 2021); however, the response of cherry rootstocks to waterlogging stress has been less studied and the underlying mechanisms remain uncharacterized. In southern China, most regions were located in the subtropical monsoon climate zone where there is more rainfall in the plum rain season, in addition to the impact of climate change, floods have occurred more frequently in recent years. In 2016, the provinces of Jiangsu, Anhui, Henan, Hubei, Jiangxi, Hunan, Sichuan, Guizhou, Yunnan, and Guangxi were severely affected by floods (Lyu et al. 2018). The roots of sweet cherries require high air permeability, and under waterlogging, the oxygen deficit leads to root rotting, gum flowing, and even death of the entire tree (Liao et al., 2011). Therefore, selecting waterlogging-tolerant rootstocks is critical for successfully cultivating sweet cherries and developing the cherry industry in southern China.

To understand how cherry rootstocks respond to waterlogging stress and whether oxygenation affects this process, we measured their relative water content (RWC), photosynthetic and chlorophyll fluorescence parameters, and antioxidant enzyme activities. Moreover, RNA-Seq analysis was performed to identify candidate genes involved in energy production, phytohormone metabolism, and encoding antioxidant enzymes and other stress-related proteins. Furthermore, we evaluated five cherry rootstock varieties under waterlogging conditions, aiming to select waterlogging-tolerant rootstocks for sweet cherry industry. We analyzed RNA-Seq data to obtain novel insights into the underlying waterlogging response mechanism of cherry rootstocks to explore critical genes and develop molecular markers, which will provide an important foundation for breeding waterlogging-tolerant cherry rootstocks.

## Results

### Precipitation conditions of 15 cherry production regions of China

**Fig. 1:**
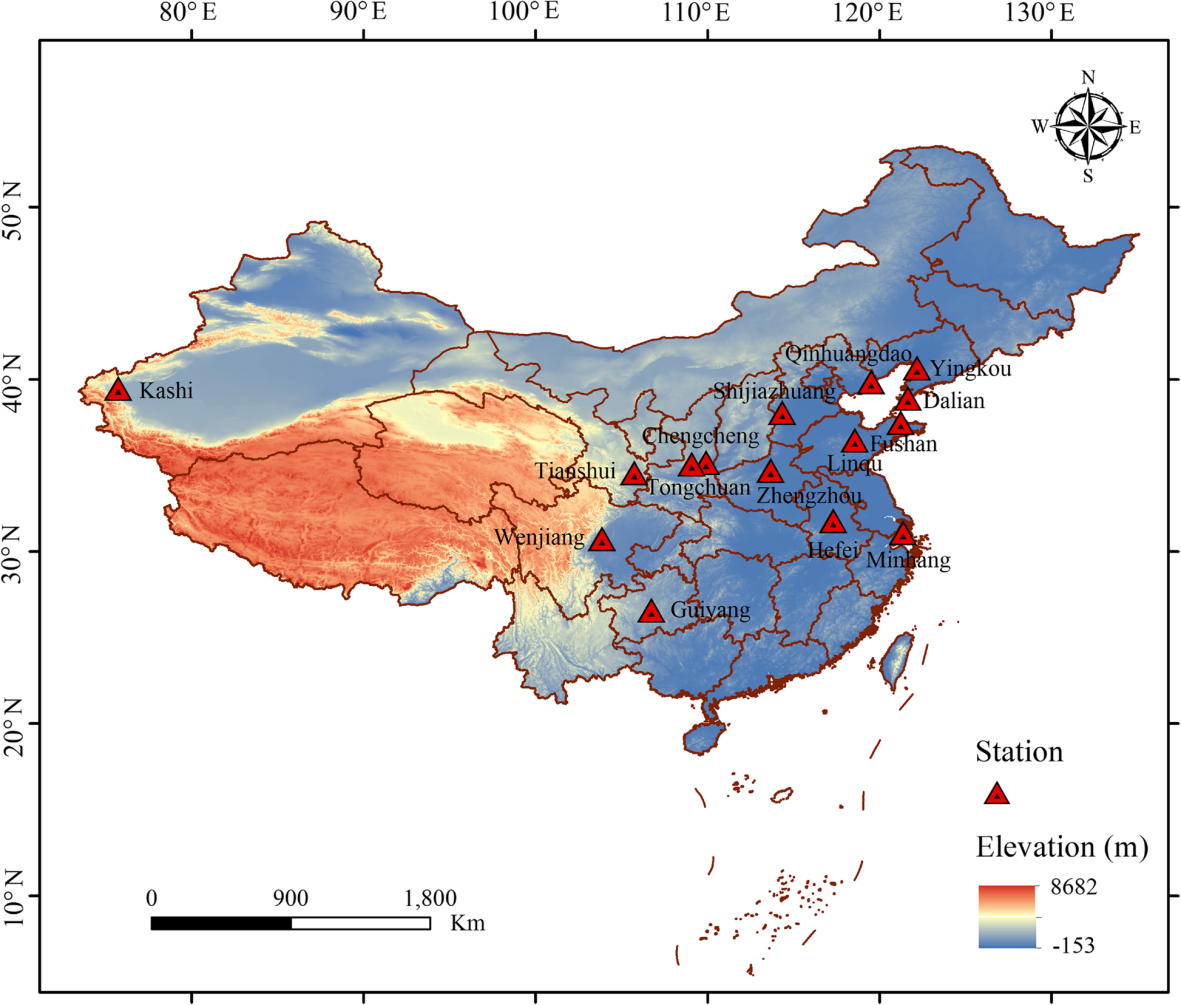
Location of 15 weather stations used as sources of primary information for assessment of past precipitation conditions. The study area colored with elevation

**Fig. 2:**
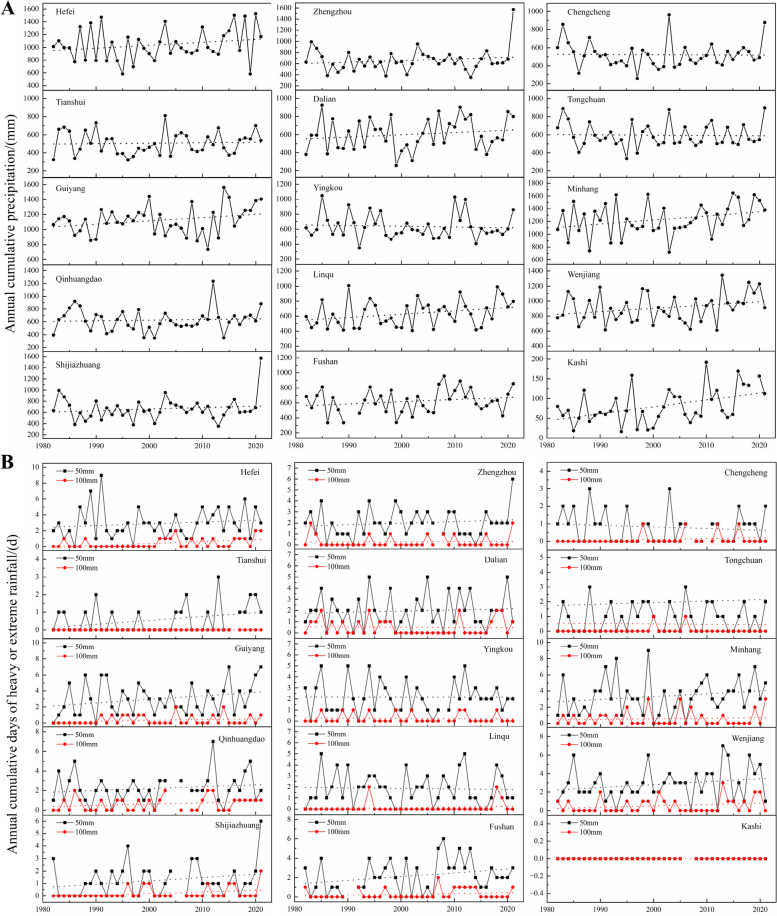
Time series of annual cumulative precipitation (**A**) and the numbers of heavy or extreme rainfall events (**B**) for 15 stations. Observations are missing from several stations for some years. Dotted lines: the linear trends from 1982–2021

Floods triggered by exceptionally heavy rainfalls are occurring at a higher frequency and negatively impact the yield and quality of crops (Hirabayashi et al. 2013; Tanoue et al. 2016; Gong et al., 2000). Therefore, we obtained precipitation data (1982–2021) from 15 stations in Chinese sweet cherry production regions to investigate the trends of cumulative precipitation and changes in the frequency of severe rainfall event occurrences in the last 40 years (Fig. 1; Table 1). Regarding the overall precipitation conditions of the 15 stations, we calculated the annual cumulative precipitation and their linear trends according to the monthly precipitation statistics (Fig. 2A). The annual cumulative precipitation of Hefei, Guiyang, Dalian, Linqu, Fushan, Minhang, Wenjiang and Kashi are on a clear upward trend; Tianshui, Qinhuangdao, Shijiazhuang and Zhengzhou are basically unchanged; while Yingkou, Chengcheng and Tongchuan are trending downward. Long-term data show the number of days of heavy (≥ 50 mm∙day^−1^) and extreme (≥ 100 mm∙day^−1^) rainfall events over China (Luo et al. 2020; Fig. 2B). Because Tianshui did not have rainfall above 100 mm and Kashi did not have rainfall above 50 mm, the corresponding position in Fig. 1 is a line with a zero value. The days of heavy rainfall within a year in Hefei, Tianshui, Guiyang, Qinhuangdao, Shijiazhuang, Fushan, Minhang and Wenjiang trended upward; Zhengzhou, Dalian, and Tongchuan remained almost constant; whereas Yingkou, Linqu, and Chengcheng trended downward. The days of extreme rainfall trends were similar to those of heavy rainfall in many stations, except for Chengcheng. In conclusion, over half of the sweet cherry production regions have increased precipitation and more frequent heavy or extreme rainfall in the last 40 years, which likely cause flooding conditions and pose risks to the yields and qualities of sweet cherries. Therefore, much attention should be focused on the mechanism of waterlogging tolerance of cherry for the better production.

**Table 1:** Weather stations used as primary sources of precipitation data in China

Province	Weather station name	Geographic coordinates		Elevation (m)
		**Latitude (°N)**	**Longitude (°E)**	
Anhui	Hefei	31.78	117.30	27.0
Gansu	Tianshui	34.57	105.74	1149.8
Guizhou	Guiyang	26.59	106.73	1223.8
Hebei	Qinhuangdao	39.85	119.52	2.4
Hebei	Shijiazhuang	38.07	114.35	103.6
Henan	Zhengzhou	34.71	113.66	110.4
Liaoning	Dalian	38.91	121.64	91.5
Liaoning	Yingkou	40.67	122.17	3.8
Shandong	Linqu	36.47	118.56	149.5
Shandong	Fushan	37.48	121.23	53.9
Shaanxi	Chengcheng	35.18	109.92	679.1
Shaanxi	Tongchuan	35.08	109.07	978.9
Shanghai	Minhang	31.10	121.37	5.5
Sichuan	Wenjiang	30.75	103.86	547.7
Xinjiang	Kashi	39.49	75.75	1385.6

### Morphological and physiological responses of five cherry rootstocks under waterlogging stress

**Fig. 3:**
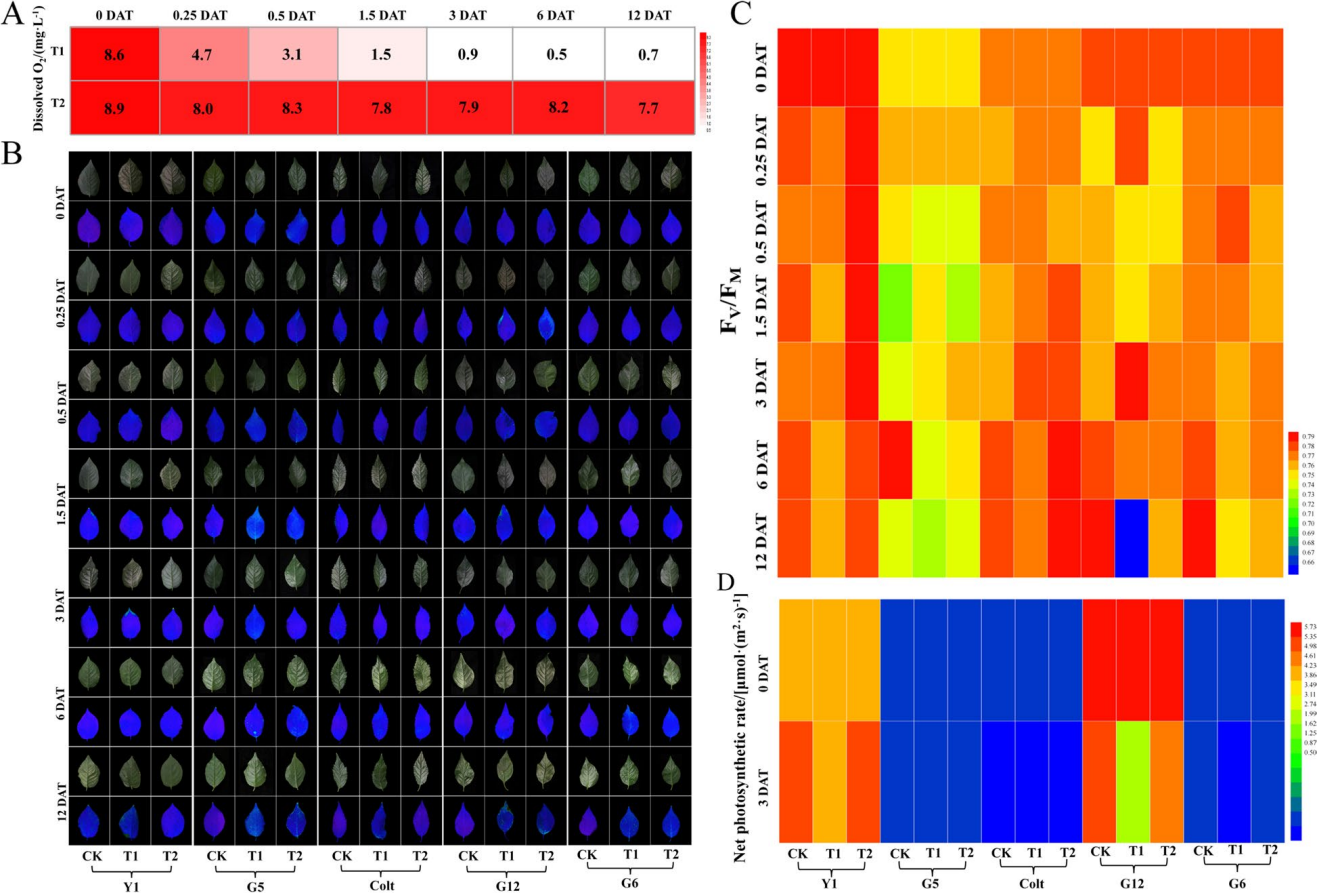
Changes in dissolved O_2_ (**A**), chlorophyll fluorescence parameters (**B** and **C**), and net photosynthetic rate (**D**) in five cherry rootstocks under waterlogging conditions, with or without oxygenation. Changes in dissolved O_2_ (**A**), chlorophyll fluorescence parameters (**B**), and Fv/Fm (**C**) were measured at 0, 0.25, 0.5, 1.5, 3, 6, and 12 days after treatment (DAT). Changes in net photosynthetic rate (**D**) were measured at 0 and 3 DAT. Bar = 3 cm

Oxygenation slowed the decrease in dissolved oxygen content under waterlogging conditions with oxygenation (T2) compared with that only subjected to waterlogging (T1) (Fig. 3A) ‘G12’ and ‘Colt’ were the most sensitive and tolerant to waterlogging among the five cherry rootstock varieties, respectively. Oxygenation alleviated the effects of waterlogging for all varieties. No morphological changes were evident within 3 d after treatment in any of the five varieties (Figure S1). Leaves of ‘G12’ under T1 began to wither and turn yellow at 6 DAT, but those of the CK and T2 did not. At 12 DAT, all the leaves of ‘G12’ under T1 were badly curled, even a gentle touch led to their abscission, indicating that it was the most waterlogging-sensitive one among the selected varieties; however, in T2, leaves of ‘G12’ were only wilted slightly. Under T1, ‘Y1’, ‘G5’, and ‘G6’ exhibited mild morphological changes, such as wilted leaves at 12 DAT. The performance of ‘Colt’ was almost the same as that prior to the waterlogging treatment, indicating that ‘Colt’ was more tolerant to waterlogging stress than the other varieties. Oxygenation reduced the damage caused by waterlogging, as the seedlings of all five varieties performed better under T2 at all sampling points. We also recorded the root phenotype at 12 DAT and found that many distal parts of the roots in all varieties turned black under T1, whereas those in the CK were unaffected (Figure S1). The percentage of roots turning black under T2 was lower than that under T1, indicating that oxygenation alleviated the stress on roots caused by waterlogging.

We determined the net photosynthetic rate (Pn) of all five varieties at 0 and 3 DAT to assess the effects of waterlogging stress on photosynthesis, with or without oxygenation, which is vital to plant growth and development (Fig. 3D). The Pn under T1 was lower than that under CK for all varieties, whereas Pn under T2 was intermediate between those under T1 and CK. ‘G12’ and ‘G6’ suffered the most significant reduction (32 and 51%, respectively) in Pn under T1, whereas ‘Y1’ exhibited a slight decrease (13%). Oxygenation greatly alleviated the effect of waterlogging in ‘Y1’ and ‘G5’, as shown by a slight difference in Pn between T2 and CK.

Changes in photosynthesis under waterlogging were assessed for the five cherry rootstocks using chlorophyll fluorescence from fresh leaves at all sampling points and the Fv/Fm ratios were calculated (Fig. 3B-C). The Fv/Fm ratio decreased for all five varieties under T1 at 6 DAT. ‘G12’ suffered the most drastic reduction in Fv/Fm under T1 at 12 DAT, consistent with the abovementioned phenotypic change and decrease in Pn, indicating that ‘G12’ was the most waterlogging-sensitive variety. Under T2, ‘Y1’ and ‘Colt’ had an Fv/Fm ratio closest to that under the control at 6 and 12 DAT, respectively, indicating that oxygenation significantly affected these two cultivars.

### Morphological, physiological, and biochemical responses of ‘G6’ under waterlogging stress

**Fig. 4:**
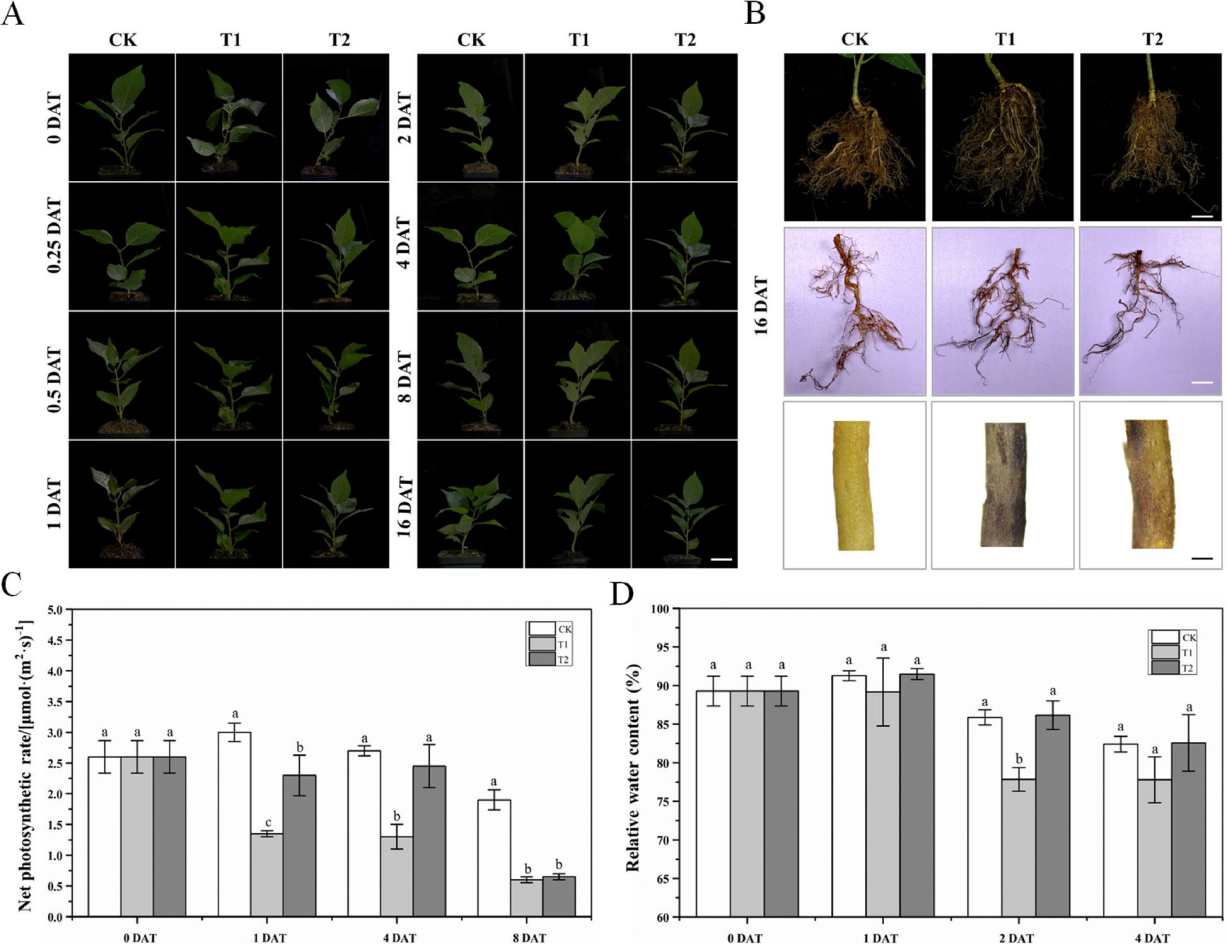
Phenotypic traits of aboveground (**A**) and underground (**B**) parts, and changes in net photosynthetic rate (**C**) and relative water content (RWC) (**D**) in ‘Gisela 6’ (‘G6’) under waterlogging conditions, with or without oxygenation. The data are shown as mean ± standard error (SE) of six replicates. Statistical significance was determined using one-way ANOVA; significant differences among means (least significant difference (LSD), *p* < 0.05) are indicated with different lowercase letters. Bars indicate 5 cm (Fig. 4A) and 3 cm, 1 cm and 1 mm (Fig. 4B), respectively

No obvious morphological changes were observed in the aerial parts of ‘G6’ throughout the experiment (Fig. 4A), indicating tolerance to waterlogging. The underground parts were photographed using a stereomicroscope to assess morphological changes at 16 DAT (Fig. 4B). Roots under T1 turned black, whereas roots under T2 only partially turned black, suggesting the mitigating effect of oxygenation. The RWC of leaves was recorded at 0, 1, 2, and 4 DAT (Fig. 4D). Waterlogging decreased the RWC of ‘G6’ seedlings at all sampling points, but the reduction was significant only at 2 DAT.

**Fig. 5:**
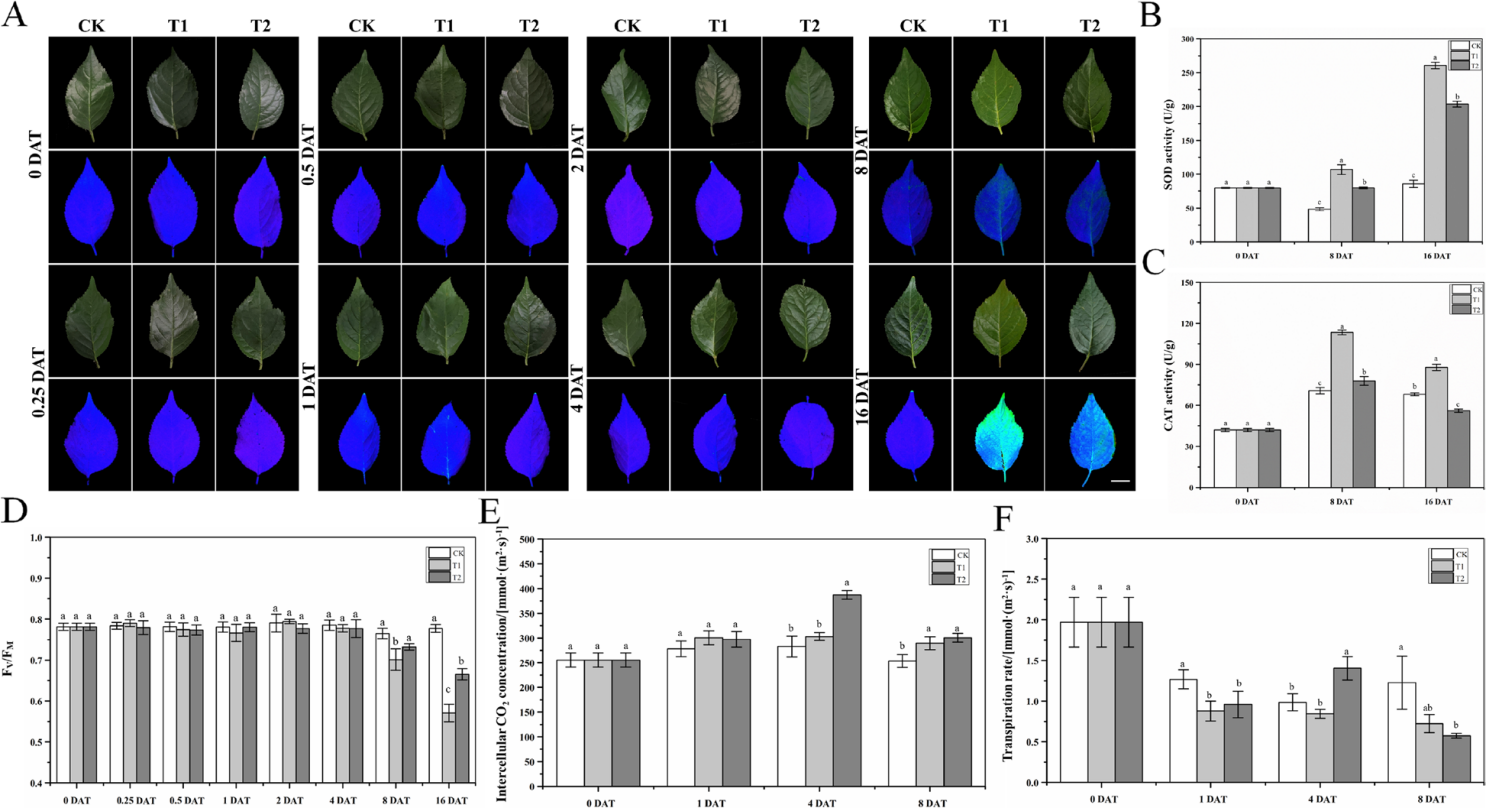
Changes in chlorophyll fluorescence parameters (**A** and **D**), antioxidant enzyme activities (**B**-**C**), and photosynthetic parameters (**E**–**F**) in ‘G6’ under waterlogging conditions, with or without oxygenation. Changes in chlorophyll fluorescence parameters (**A**) and Fv/Fm (**D**) were measured at 0, 0.25, 0.5, 1, 2, 4, 8 and 16 DAT. Superoxide dismutase (SOD) (**B**) and catalase (CAT) (**C**) activities were measured at 0, 8, and 16 DAT. Intercellular CO_2_ concentration (**E**) and transpiration rate (**F**) were measured at 0, 1, 4 and 8 DAT. The data are shown as mean ± standard error (SE) of six replicates. Statistical significance was determined using a one-way ANOVA; significant differences among means (LSD, *p* < 0.05) are indicated with different lowercase letters. Bar = 3 cm

The Pn of ‘G6’ seedlings was determined at 0, 1, 4, and 8 DAT (Fig. 4C). The Pn under T1 was lower than that under CK at 1 DAT, and the Pn under T2 was intermediate between that under T1 and CK at all harvest points. Oxygenation significantly enhanced the Pn of ‘G6’ under waterlogging stress at 1 and 4 DAT (*p* < 0.05). However, the effect of oxygenation on Pn was not distinct at 8 DAT under both T1 and T2 showing drastic reductions. The intercellular CO_2_ concentration and transpiration rate displayed irregular trends under the three treatments at 0, 1, 4, and 8 DAT (Fig. 5E-F). At 1 DAT, the intercellular CO_2_ concentrations under T1 and T2 were not significantly different from that under CK. However, at 4 DAT, the CO_2_ concentration under T2 was the highest, whereas it remained unchanged in the leaves under T1. The burst of intercellular CO_2_ concentrations under T2 at 4 DAT did not last long, and little difference was observed under T1 and T2 at 8 DAT, although the concentrations under these treatments were significantly different from those under CK. The changes in the transpiration rate under different treatments were more drastic. At 1 DAT, the transpiration rates under T1 and T2 were lower than those under CK, although the difference between the two treatments was not significant. Interestingly, the transpiration rate under T2 also showed a burst at 4 DAT, which was the highest of the three treatments. However, at 8 DAT, the burst disappeared, and the transpiration rate under T2 was remarkably lower than that under CK simultaneously, the transpiration rate under T1 was not significantly different from that under CK and T2.

To further investigate the changes in photosynthesis of ‘G6’ under waterlogging conditions and the recovery effect of oxygenation, we recorded the chlorophyll fluorescence of leaves and calculated the Fv/Fm ratio (Fig. 5A and D). The Fv/Fm ratio under T1 decreased at 8 DAT, whereas no significant difference was found between T2 and CK. The Fv/Fm ratio was drastically reduced under T1 at 16 DAT simultaneously, that under T2 was significantly different from that under T1 and CK, indicating that oxygenation still alleviated the effect of waterlogging stress, even at 16 DAT; however, the recovery was not comparable to that in the early stage of treatment.

There were also changes in waterlogging ‘G6’ at the biochemical level, and SOD and CAT activities were determined at 0, 8, and 16 DAT (Fig. 5B-C). At 8 DAT, ‘G6’ under T1 showed a significant increase in SOD activity compared with that under CK, whereas the activity under T2 was intermediate between that under T1 and CK but remarkably different from both. A similar pattern was observed at 16 DAT, except that SOD activities under T1 and T2 were more than double at 8 DAT. The CAT activity under T1 was higher than that under CK at 8 DAT. Although CAT activity under T2 was also significantly different from that under CK at 8 DAT, this difference was not obvious. However, at 16 DAT, the CAT activity was reduced under T1 and T2. The CAT activity was the highest under T1, followed by CK and then T2.

### Significant differentially expressed genes (DEGs) identified at 8 DAT after waterlogging stress (T1) and oxygenating (T2) treatments

Transcriptome analysis was performed to investigate the genes involved in waterlogging stress response. Nine cDNA libraries were constructed using ‘G6’ leaves collected at 8 DAT from the T1, T2, and CK treatments. A total of 421,344,742 raw reads were obtained and the raw data were uploaded to the NCBI Sequence Read Archive (SRA) database (accession number PRJNA866188). After filtering the data, clean reads ranging from 37,029,532 to 45,786,676, with an average of 39,994,256 were obtained for each cDNA library. The Q20 for clean reads was above 96.03%. An overview of sequence data analysis is presented in Table S2. Among the 359,948,306 clean reads, 311,517,174 (86.54%) were mapped and 295,306,906 (82.04%) were uniquely mapped onto the cherry genome; the percentage of multiple mapped reads was less than 4.50% (Table S3). Pearson’s correlation analysis revealed a good correlation among the three biological replicates (Figure S2). The abovementioned information indicates the high quality and accuracy of RNA-seq data, which was required for subsequent analyses.

We focused on the DEGs identified by comparing the gene expression levels between the T1 and T2 treatments and CK to explore candidate genes modulated in response to waterlogging stress and oxygenation. Comprehensive gene expression profiles are shown as circles (Figure S3) and heatmaps (Figure S4A). A total of 893 and 414 genes were upregulated and 651 and 275 genes were downregulated in the samples from the T1 and T2 treatments, respectively, compared with CK, whereas 187 genes were upregulated and 259 genes were downregulated under T2 compared to those under T1 (Figure S4B). The DEGs induced under T1 were considerably more than those under T2, suggesting the waterlogging stress-relieving effect of oxygenation. The Venn diagram presents unique and common DEGs in ‘G6’ under the different treatments (Figure S4C).

### Identification of cellular and molecular events involved in waterlogging stress response with or without oxygenation using GO and KEGG analysis

Gene ontology (GO) enrichment analysis was performed to explore the biological functions of DEGs. In the comparison between CK and T1 (CK *vs*. T1), the highly enriched GO terms were “photosynthesis” in the biological process (BP), “catalytic activity” and “oxidoreductase activity” in the molecular function (MF), and “membrane” and “thylakoid” in the cellular component (CC) (Figure S5A-B). For CK *vs*. T2, the highly enriched GO terms were “DNA-binding transcription factor activity” and “transcription regulator activity” in the MF, “membrane” and “cellular anatomical entity” in the CC, and “response to bacterium” and “defense response to bacterium” in the BP (Figure S5C-D). For T2 *vs*. T1, the highly enriched GO terms were “calcium iron binding” and “carbohydrate binding” in the MF, “response to inorganic substance” and “response to water” in the BP, and “dynein complex” in the CC (Figure S5E-F).

KEGG pathway enrichment analysis for DEGs was performed to identify biological pathways. It revealed that 358 of the 4,540 DEGs between the T1 treatment and CK were enriched in 95 pathways (Table S4). In the waterlogging treatment, the top five enriched pathways were photosynthesis-antenna proteins (pavi00196), photosynthesis (pavi00195), glutathione metabolism (pavi00480), alanine, aspartate and glutamate metabolism (pavi00250), and galactose metabolism (pavi00052) (Figure S6A-B). Moreover, 141 of the 3120 DEGs between the T2 treatment and CK were enriched in 53 pathways (Table S5). In the T2 treatment, the top five enriched pathways were glutathione metabolism (pavi00480), galactose metabolism (pavi00052), photosynthesis-antenna proteins (pavi00196), biosynthesis of various secondary metabolites-part 3 (pavi00997), and alanine, aspartate and glutamate metabolism (pavi00250) (Figure S6C-D). In T2 *vs*. T1, the top five enriched pathways were nitrogen metabolism (pavi00910), ascorbate and aldarate metabolism (pavi00053), sesquiterpenoid and triterpenoid biosynthesis (pavi00909), zeatin biosynthesis (pavi00908), and amino sugar and nucleotide sugar metabolism (pavi00520) (Figure S6E-F) A total of 81 of the 2,379 DEGs between the T2 and T1 treatments were enriched in 41 pathways (Table S6). These annotations helped us better understand the adaptive responses to waterlogging in cherry rootstocks.

### DEGs related to energy production

**Fig. 6:**
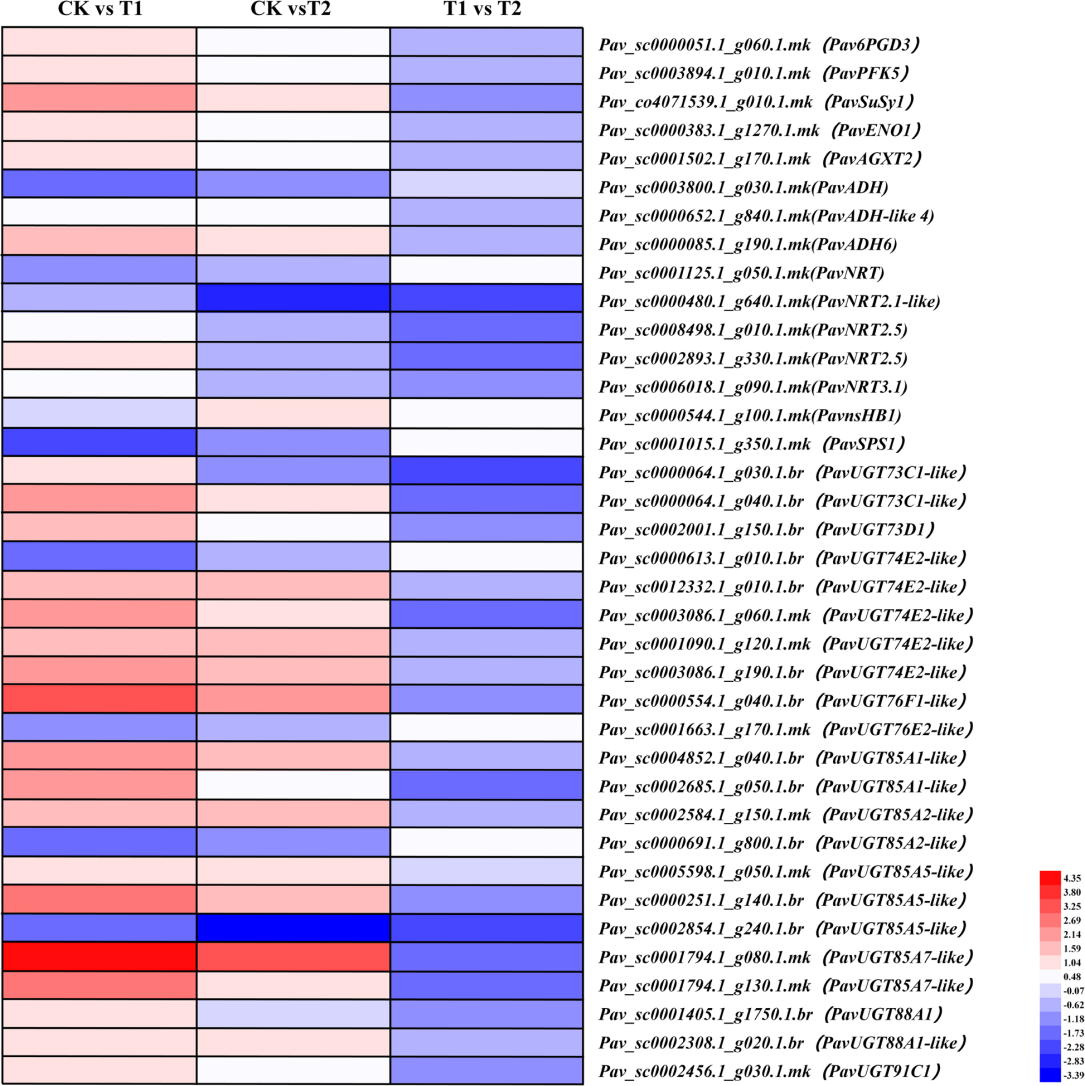
Expression profiles of differentially expressed genes related to energy production, shown using a heatmap. The scale of color intensity is in the lower right quarter of heatmap, representing the log_2_fold-change values. Fold-change refers to the ratio of gene expression level in cherry rootstock leaves between control (CK) and treatments (T1/T2)

The glycolysis and fermentation pathways are activated in response to low oxygen conditions, therefore, the enzymes involved in these pathways were investigated (Fig. 6). The glycolytic enzymes 6-phosphogluconate dehydrogenase (*Pav6PGD3*), 6-phosphofructokinase (*PavPFK5*), and enolase (*PavENO1*) were significantly induced under waterlogging conditions (Table S7). These three genes were also activated under T2, but the increase in expression was gentler than that under T1. Alcohol dehydrogenase (*PavADH*, *PavADH-like 4*, *PavADH6*) is a critical enzyme involved in ethanol fermentation, *PavADH-like 4* (*Pav_sc0000652.1_g840.1.mk*) and *PavADH6* (*Pav_sc0000085.1_g190.1.mk*) were induced under T1 and T2, whereas *PavADH* (*Pav_sc0003800.1_g030.1.mk*) was suppressed under both treatments. Sucrose synthase (*PavSuSy1*), which plays a critical role in maintaining sugar supply under waterlogging (Kumutha et al., 2008), was highly activated in the waterlogged plants. Alanine-glyoxylate aminotransferase (*PavAGXT2*), which functions in the regeneration of pyruvate, a product of glycolysis, was upregulated under T1 and T2.

Other carbohydrate synthesis and metabolism genes, including UDP-glycosyltransferase (*PavUGTs*) and sucrose-phosphate synthase 1 (*PavSPS1*), were also analyzed. Twenty-two *PavUGTs* were identified, among which *PavUGT74E2-like* (*Pav_sc0000613.1_g010.1.br*), *PavUGT76E2-like* (*Pav_sc0001663.1_g170.1.mk*), *PavUGT85A2-like* (*Pav_sc0000691.1_g800.1.br*), and *PavUGT85A5-like* (*Pav_sc0002854.1_g240.1.br*) were downregulated after waterlogging, whereas 18 transcripts were highly induced.

Plant mitochondria can synthesize ATP under anoxic conditions using NADH and NADPH as electron donors and nitrite as an electron acceptor, substituting oxygen in the respiratory chain (Stoimenova et al. 2007). Five DEGs encoding nitrate transporters (NRTs), which are used to absorb nitrate from the soil, were identified in cherry rootstock leaves, among which *PavNRT* (*Pav_sc0001125.1_g050.1.mk*) and *PavNRT2.1-like* (*Pav_sc0000480.1_g640.1.mk*) were downregulated after both treatments, whereas *PavNRT2.5* (*Pav_sc0008498.1_g010.1.mk*, and *Pav_sc0002893.1_g330.1.mk*) and PavNRT3.1 (*Pav_sc0006018.1_g090.1.mk*) were activated under T1, but suppressed under T2, indicating different functions for different NRTs. Denitrification benefits plants, but it also leads to the production of nitric oxide (NO), which is a toxic byproduct (Huang et al. 2002). Non-symbiotic hemoglobins (nsHBs) scavenge NO. Perazzolli et al. (2004) reported that Arabidopsis *AtnsHB1* produces S-nitrosohemoglobin and decreases NO emission under low oxygen conditions. Here, *PavnsHB1* (*Pav_sc0000544.1_g100.1.mk*) was induced under both treatments.

### Expression profiles of genes involved in ethylene (ETH) biosynthesis and signal transduction

**Fig. 7:**
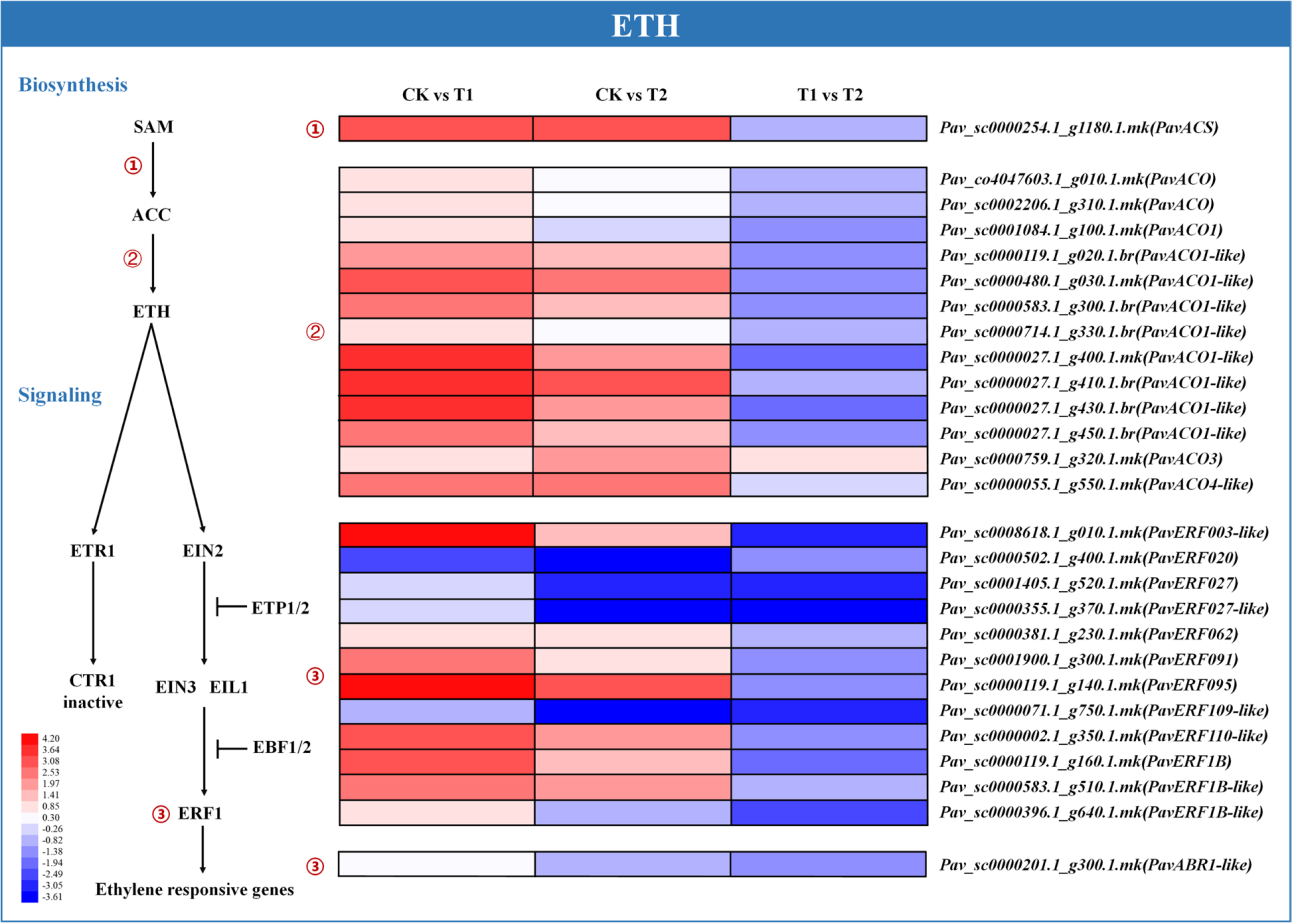
Expression profiles of differentially expressed genes (DEGs) related to ethylene (ETH) biosynthesis and signaling pathways, represented using a heatmap. The scale of color intensity is shown in the lower left quarter of heat map representing the log2fold-change values. Fold-change refers to the ratio of gene expression levels in cherry rootstock leaves between control (CK) and treatments (T1/T2)

The expression profiles of genes associated with phytohormone metabolic pathways were investigated. The expression levels of DEGs associated with ethylene biosynthesis, signal transduction and deactivation were analyzed (Fig. 7 and Table S8). Representative genes included ETH synthase genes 1-aminocyclopropane-1-carboxylate synthase (*PavACS*), 1-aminocyclopropane-1-carboxylate oxidase (*PavACO*, *PavACO1*, *PavACO1-like*, *PavACO3*, and *PavACO4-like*), and genes involved in ETH signal transduction, ethylene-responsive transcription factor ABR1-like (*PavABR1-like*) and ethylene-responsive transcription factor (*PavERFs*). *PavACO* (*Pav_co4047603.1_g010.1.mk*, and *Pav_sc0002206.1_g310.1.mk*), *PavACO1* (*Pav_sc0001084.1_g100.1.mk*), *PavACO1-like* (*Pav_sc0000119.1_g020.1.br*, *Pav_sc0000480.1_g030.1.mk*, *Pav_sc0000583.1_g300.1.br*, *Pav_sc0000714.1_g330.1.br*, *Pav_sc0000027.1_g400.1.mk*, *Pav_sc0000027.1_g410.1.br*, *Pav_sc0000027.1_g430.1.br*, and *Pav_sc0000027.1_g450.1.br*), *PavACO3* (*Pav_sc0000759.1_g320.1.mk*), and *PavACO4-like* (*Pav_sc0000055.1_g550.1.mk*) were highly upregulated under T1, but only *PavACO1-like* (*Pav_sc0000119.1_g020.1.br*, *Pav_sc0000480.1_g030.1.mk*, *Pav_sc0000583.1_g300.1.br*, *Pav_sc0000027.1_g400.1.mk*, and *Pav_sc0000027.1_g410.1.br*), *PavACO3* (*Pav_sc0000759.1_g320.1.mk*), and *PavACO4-like* (*Pav_sc0000055.1_g550.1.mk*) were significantly induced under T2. In addition, the expression of *PavACO1* (*Pav_sc0001084.1_g100.1.mk*) and *PavACO1-like* (*Pav_sc0000583.1_g300.1.br*, and *Pav_sc0000027.1_g400.1.mk*) was remarkably repressed under T2 compared with that under T1, indicating the effect of oxygenation. *PavACS* (*Pav_sc0000254.1_g1180.1.mk*) was remarkably activated under both treatments, but there was no significant difference between the two treatments. *PavABR1-like* (*Pav_sc0000201.1_g300.1.mk*) was upregulated under T1, but slightly downregulated under T2. Among ETH signal transduction genes, *PavERF003-like* (*Pav_sc0008618.1_g010.1.mk*), *PavERF062* (*Pav_sc0000381.1_g230.1.mk*), *PavERF091* (*Pav_sc0001900.1_g300.1.mk*), *PavERF095* (*Pav_sc0000119.1_g140.1.mk*), *PavERF110-like* (*Pav_sc0000002.1_g350.1.mk*), *PavERF1B* (Pav_sc0000119.1_g160.1.mk), and *PavERF1B-like* (*Pav_sc0000583.1_g510.1.mk*, and *Pav_sc0000396.1_g640.1.mk*) were significantly induced under T1, whereas only *PavERF095* (*Pav_sc0000119.1_g140.1.mk*), *PavERF1B* (*Pav_sc0000119.1_g160.1.mk*), and *PavERF1B-like* (*Pav_sc0000583.1_g510.1.mk*) were significantly activated under T2. However, *PavERF020* (*Pav_sc0000502.1_g400.1.mk*) was remarkably repressed under both treatments, unlike other *PavERFs*.

### Expression profiles of genes involved in abscisic acid (ABA) biosynthesis and signal transduction

**Fig. 8:**
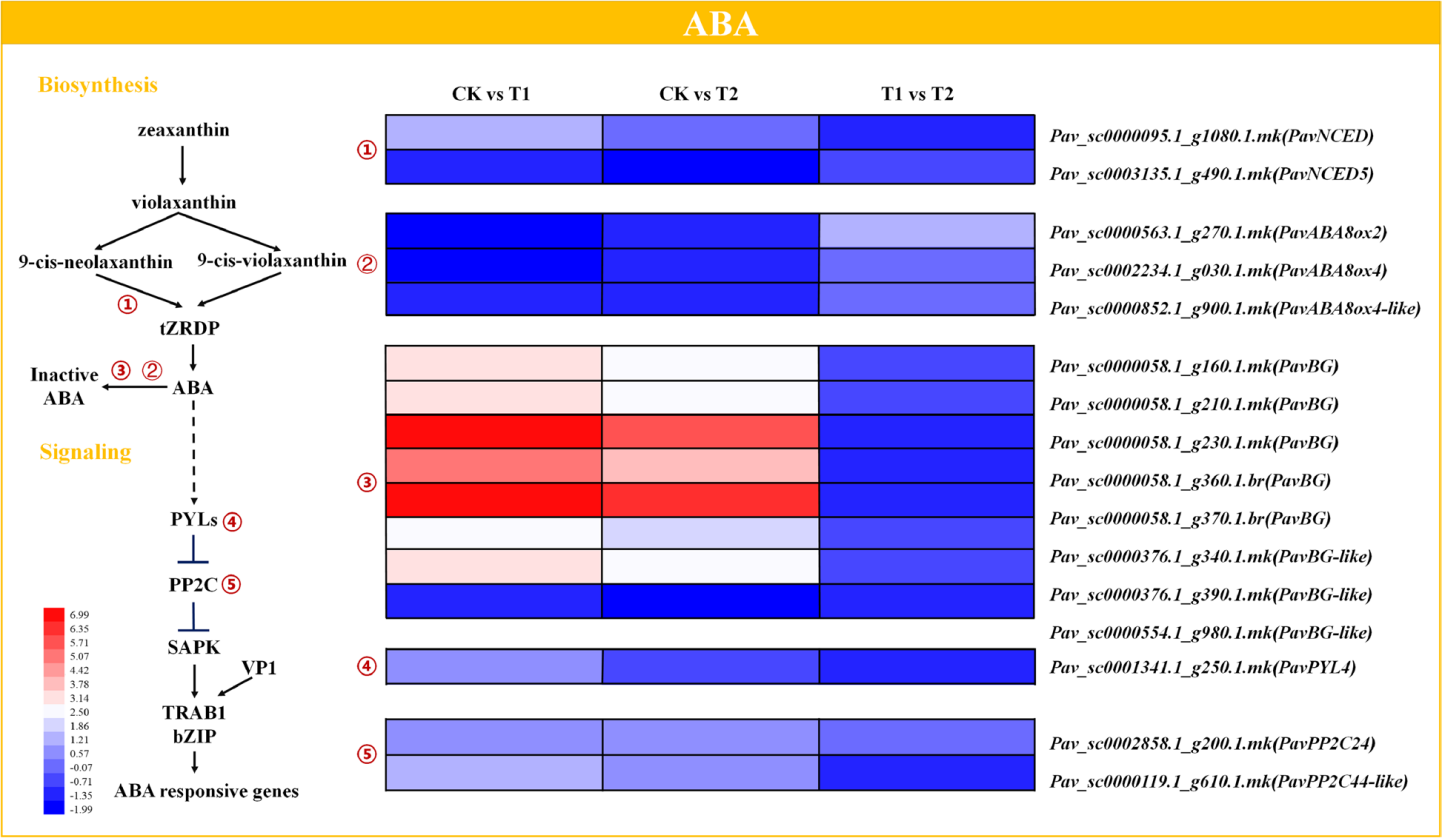
Expression profiles of differentially expressed genes related to ABA biosynthesis and signaling pathways, shown using a heatmap. The scale of color intensity is in the lower left quarter of heatmap, representing the log_2_fold-change values. Fold-change refers to the ratio of gene expression level in cherry rootstock leaves between control (CK) and treatments (T1/T2)

DEGs encoding proteins involved in ABA biosynthesis, signal transduction and deactivation were identified (Fig. 8 and Table S9). The representative genes involved were ABA synthase genes *NCED* (*PavNCED* and *PavNCED5*), ABA receptor *PYL* (*PavPYL4*), ABA signaling gene protein phosphatase 2Cs (*PavPP2C24* and *PavPP2C44-like*), ABA metabolism genes *ABA8ox* (*PavABA8ox2*, *PavABA8ox4*, and *PavABA8ox4-like*), and beta-glucosidase (*PavBG* and *PavBG-like*). The expression of *PavNCED* (*Pav_sc0000095.1_g1080.1.mk*) was significantly activated in response to waterlogging, whereas *PavNCED5* was significantly repressed under both treatments. The *ABA8ox* genes (*Pav_sc0000563.1_g270.1.mk*, *Pav_sc0002234.1_g030.1.mk*, and *Pav_sc0000852.1_g900.1.mk*) were significantly inhibited under T1, but insignificantly under T2. *Pav_sc0000563.1_g270.1.mk* was significantly upregulated under T2 compared to T1, demonstrating that the critical enzymes in ABA degradation, *PavABA8oxs*, played important roles in waterlogging tolerance, and oxygenation alleviated the effect of waterlogging. Most *PavBGs* (*Pav_sc0000058.1_g160.1.mk*, *Pav_sc0000058.1_g210.1.mk*, *Pav_sc0000058.1_g230.1.mk*, *Pav_sc0000058.1_g360.1.br*, *Pav_sc0000058.1_g370.1.br*, *Pav_sc0000376.1_g340.1.mk*, and *Pav_sc0000376.1_g390.1.mk*) were significantly induced under both treatments, except for *Pav_sc0000554.1_g980.1.mk*, which was repressed after both treatments. *PavPYL4* (*Pav_sc0001341.1_g250.1.mk*), encoding the abscisic acid receptor PYL4, was upregulated under T1 but was slightly inhibited under T2. The expression of *PavPP2Cs* (*Pav_sc0002858.1_g200.1.mk* and *Pav_sc0000119.1_g610.1.mk*) was upregulated under both treatments, indicating that *PP2C* may function in resisting waterlogging stress.

### Expression profiles of genes involved in auxin biosynthesis and signal transduction

**Fig. 9:**
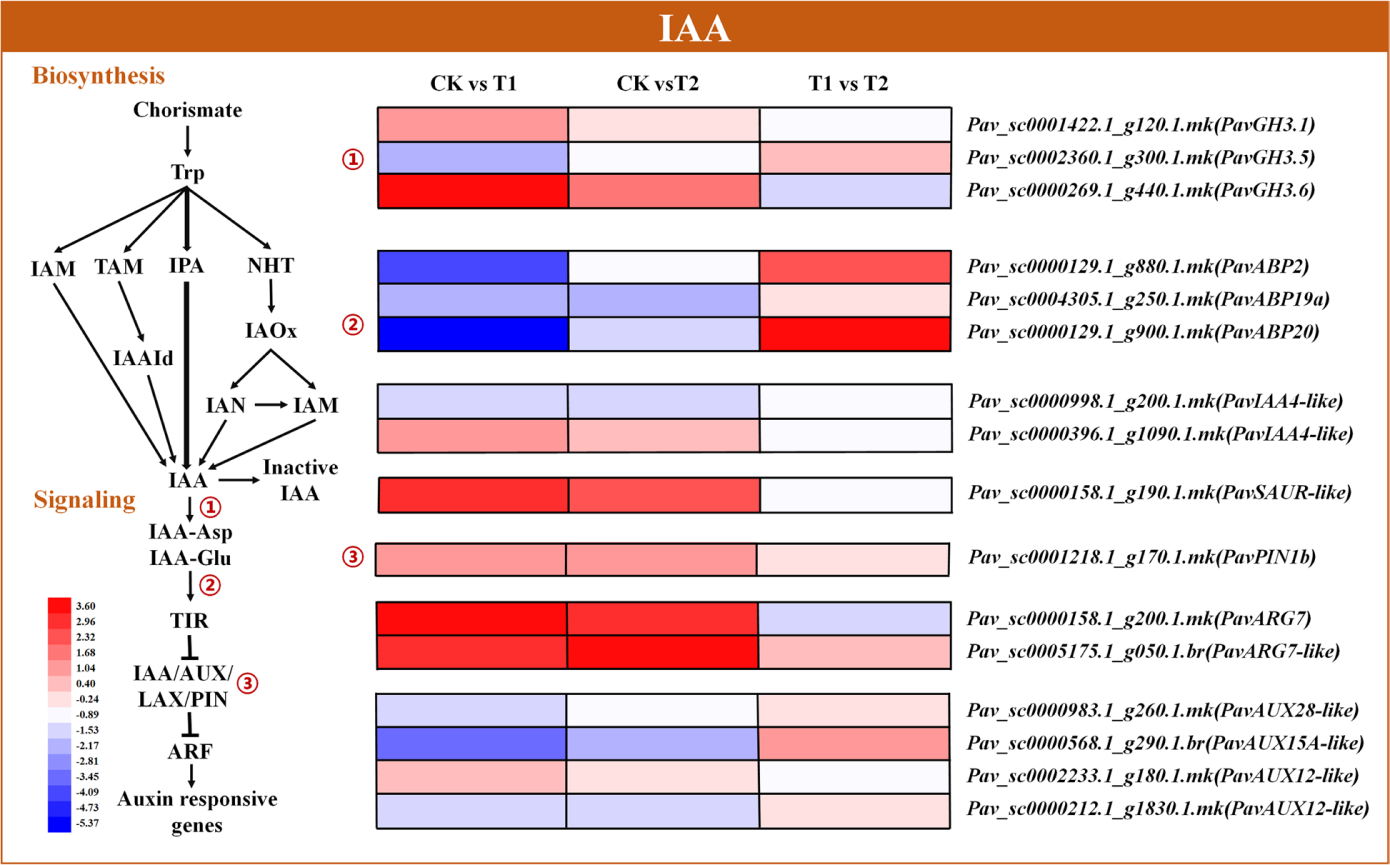
Expression profiles of DEGs related to indole-3-acetic acid (IAA) biosynthesis and signaling pathways, represented using a heatmap. The scale of color intensity is shown in the lower left quarter of heatmap, representing the log_2_fold-change values. Fold-change refers to the ratio of gene expression levels in cherry rootstock leaves between control (CK) and treatments (T1/T2)

Indole-3-acetic acid (IAA) plays an important role as the main endogenous auxin in plants, and two putative IAA synthetic pathways have been reported previously (Fig. 9 and Table S10). In the present study, the representative genes identified were IAA biosynthesis genes *indole-3-acetic acid-amido synthetase* (*PavGH3.1*, *PavGH3.5*, and *PavGH3.6*), and genes related to IAA signal transduction, including those encoding auxin-responsive proteins (*PavIAA4-like* and *PavSAUR-like*), auxin-binding proteins (*PavABP2*, *PavABP19a*, and *PavABP20*), auxin efflux carrier (*PavPIN1b*), IAA-induced proteins (*PavARG7* and *PavARG7-like*), and auxin-induced proteins (*PavAUX28-like*, *PavAUX15A-like*, *PavAUX12-like*, and *PavAUX12-like*). *PavGH3.1* (*Pav_sc0001422.1_g120.1.mk*) and *PavGH3.6* (*Pav_sc0000269.1_g440.1.mk*) were significantly upregulated under T1, whereas *PavGH3.5* (*Pav_sc0002360.1_g300.1.mk*) was repressed under both treatments. Among the auxin-responsive protein genes, *PavIAA4-like* (*Pav_sc0000396.1_g1090.1.mk*) and *PavSAUR-like* (*Pav_sc0000158.1_g190.1.mk*) were remarkably activated under T1, whereas *PavIAA4-like* (*Pav_sc0000998.1_g200.1.mk*) was restrained under both treatments. Three auxin-binding protein genes, *PavABP2* (*Pav_sc0000129.1_g880.1.mk*), *PavABP19a* (*Pav_sc0004305.1_g250.1.mk*), and *PavABP20* (*Pav_sc0000129.1_g900.1.mk*) were suppressed under both treatments. *PavPIN1b* (*Pav_sc0001218.1_g170.1.mk*), an auxin efflux carrier gene, was significantly induced under both treatments. Among auxin-induced protein genes, *PavARG7* (*Pav_sc0000158.1_g200.1.mk*), *PavARG7-like* (*Pav_sc0005175.1_g050.1.br*), and *PavAUX12-like* (*Pav_sc0002233.1_g180.1.mk*) were activated under both treatments, whereas *PavAUX28-like* (*Pav_sc0000983.1_g260.1.mk*), *PavAUX15A-like* (*Pav_sc0000568.1_g290.1.br*), and *PavAUX12-like* (*Pav_sc0000212.1_g1830.1.mk*) were repressed.

### Expression profiles of genes involved in cytokinin (CTK) biosynthesis and signal transduction

The expression levels of DEGs associated with CTK synthesis and signal transduction were analyzed (Figure S7 and Table S11). Representative genes involved CTK synthase genes cytokinin riboside 5'-monophosphate phosphoribohydrolase *LOGs* (*PavLOG1* and *PavLOG3-like*), CTK metabolism genes cytokinin dehydrogenase (*PavCKX6-like*), CTK transporters purine permease (*PavPUP11*), ABC transporter G family members (*PavABCG6-like*, *PavABCG11-like* and *PavABCG29-like*), and CTK signaling gene histidine-containing phosphotransfer protein 4-like (*PavHP4-like*). *PavLOG1* (*Pav_sc0000480.1_g080.1.mk*) and *PavLOG3-like* (*Pav_sc0000711.1_g050.1.mk*) were significantly induced under T1, and the latter was significantly activated under T2. *PavCKX6-like* (*Pav_sc0000507.1_g100.1.mk*) was upregulated under both treatments. Similarly, the CTK transporter, *PavPUP11* (*Pav_sc0001243.1_g270.1.mk*) was also significantly activated under both treatments. ABC transporter G family members identified in the cherry genome showed different expression profiles. Specifically, *PavABCG11-like* (*Pav_co4020073.1_g010.1.br* and *Pav_sc0006269.1_g020.1.br*) and *PavABCG29-like* (*Pav_sc0002842.1_g320.1.mk*) were upregulated under T1, whereas *PavABCG6-like* (*Pav_sc0000554.1_g2270.1.mk*) was repressed. Another CTK transporter gene, *PavHP4-like* (*Pav_sc0000800.1_g510.1.mk*), was significantly downregulated under T1 but the downregulation under T2 was not clear.

### Expression profiles of genes involved in gibberellin (GA), brassinosteroids (BR), and salicylic acid (SA) biosynthesis and signal transduction

The expression profiles of DEGs associated with GA biosynthesis, signal transduction and deactivation were analyzed (Figure S8 and Table S12). Terpene synthase (*PavTPS*), ent-kaurenoic acid oxidase (*PavKAO*), and gibberellin 2-beta-dioxygenase (*PavGA2ox*), which are related to GA biosynthesis and deactivation, were significantly upregulated and downregulated under both treatments. Specifically, *PavGA2ox1-like* (*Pav_sc0000095.1_g1110.1.mk*), *PavGA2ox8* (*Pav_sc0000638.1_g630.1.mk*), and *PavGA2ox8-like* (*Pav_sc0000410.1_g210.1.mk*) were remarkably activated under T1, with no clear change being observed under T2. However, *PavGA3ox* (*Pav_sc0000716.1_g300.1.mk*) was significantly repressed under both treatments. *PavTPS* (*Pav_sc0000333.1_g240.1.mk*, and *Pav_sc0000872.1_g240.1.mk*) involved in the initial steps of GA biosynthesis was remarkably downregulated under T1, but oxygenation (T2 treatment) alleviated this downregulation. *PavKAO1-like* (*Pav_sc0000503.1_g830.1.mk*), which also functions in GA biosynthesis, was significantly induced under T1.

BR and SA pathways play a role in stress tolerance. DEGs associated with BR and SA biosynthesis and signal transduction were also detected (Figure S8; Tables S13 and S14). These genes included those encoding the BR-regulated protein, *BRU1-like* (*PavBRU1-like*), SA-related genes pathogenesis-related protein, *PR-4-like* (*PavPR4-like*), and *salicylic acid-binding protein 2-like* (*PavSABP2-like*). *PavBRU1-like* (*Pav_sc0003915.1_g020.1.mk*) was significantly repressed under T1; however, the downregulation of *PavBRU1-like* under T2 was not as strong. Among the SA-related genes, *PavPR4-like* (*Pav_sc0000396.1_g1070.1.mk*) was significantly upregulated under both treatments. Similarly, two transcripts of *PavSABP2-like* (*Pav_sc0000348.1_g400.1.mk* and *Pav_sc0000348.1_g500.1.mk*) were remarkably activated under both treatments.

### DEGs related to stress-related transcription factors

**Fig. 10:**
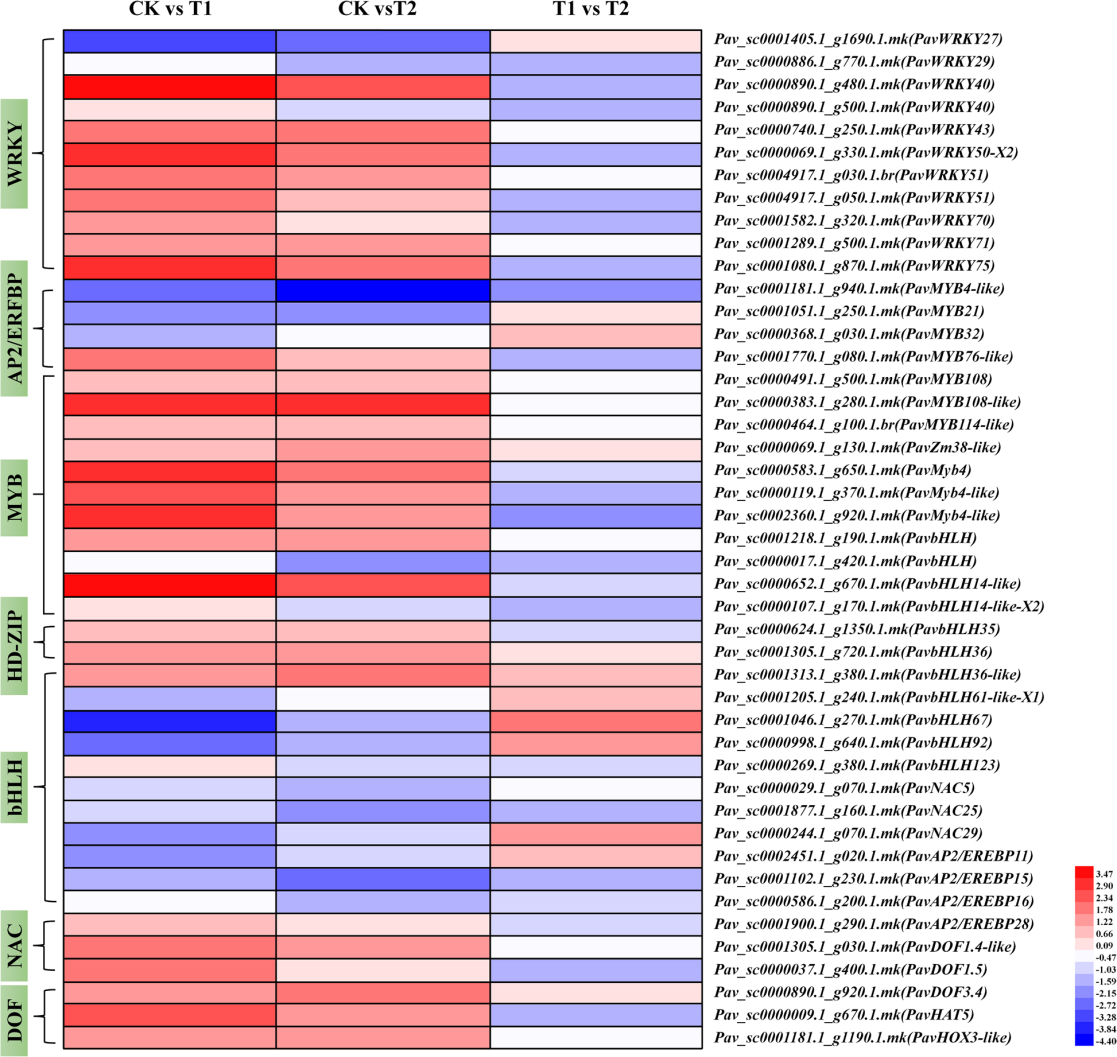
Expression profiles of DEGs related to stress associated transcription factors, shown using a heatmap. The scale of color intensity is shown in the lower left quarter of heatmap, representing the log_2_fold-change values. Fold-change refers to the ratio of gene expression levels in cherry rootstock leaves between control (CK) and treatments (T1/T2)

Transcriptional regulation of gene expression is a critical regulatory mechanism in plants and is mostly mediated through transcription factors that recognize and bind to *cis*-acting elements in the promoter and enhancer regions of the corresponding genes (Meshi and Iwabuchi 1995). The transcription factors identified in the cherry genome included WRKY, MYB, MYB-related, bHLH, NAC, AP2/ERBP, DOF, HD-ZIP and DBP (Fig. 10 and Table S15). Waterlogging (T1 treatment) significantly activated seven WRKY transcription factors (*PavWRKY40*, *PavWRKY43*, *PavWRKY50-X2*, *PavWRKY51*, *PavWRKY70*, *PavWRKY71*, and *PavWRKY75*), whereas only three were significantly induced under T2. However, *PavWRKY27* (*Pav_sc0001405.1_g1690.1.mk*) and *PavWRKY29* (*Pav_sc0000886.1_g770.1.mk*) were repressed under both treatments, whereas *PavWRKY40* (*Pav_sc0000890.1_g500.1.mk*) was induced under T1 but suppressed under T2. All seven identified MYB transcription factors (*PavMYB4-like*, *PavMYB21*, *PavMYB32*, *PavMYB76-like*, *PavMYB108*, *PavMYB108-like*, and *PavMYB114-like*) were upregulated under both treatments. Among the four MYB-related transcription factors, *PavZm38-like* (*Pav_sc0000069.1_g130.1.mk*) and *PavMyb4-like* (*Pav_sc0000119.1_g370.1.mk*) were significantly activated under both treatments. *PavMyb4* (*Pav_sc0000583.1_g650.1.mk*) was repressed under both treatments, whereas *PavMyb4-like* (*Pav_sc0002360.1_g920.1.mk*) was upregulated under T1, and suppressed under T2. Nine bHLH transcription factors (*PavbHLH*, *PavbHLH14-like*, *PavbHLH14-like-X2*, *PavbHLH36*, *PavbHLH36-like*, *PavbHLH61-like-X1*, *PavbHLH67*, *PavbHLH92*, and *PavbHLH123*) were downregulated under both treatments, whereas *PavbHLH* (*Pav_sc0001218.1_g190.1.mk*) was activated under both treatments. *PavbHLH35* (*Pav_sc0000624.1_g1350.1.mk*) was slightly induced under T1 and repressed under T2. Three NAC transcription factors (*PavNAC5*, *PavNAC25*, and *PavNAC29*) were significantly upregulated under T1, and *PavNAC25* (*Pav_sc0001877.1_g160.1.mk*) was significantly induced under T2. Three of the four identified AP2/EREBP transcription factors, *PavAP2/EREBP11* (*Pav_sc0002451.1_g020.1.mk*), *PavAP2/EREBP15* (*Pav_sc0001102.1_g230.1.mk*), and *PavAP2/EREBP16* (*Pav_sc0000586.1_g200.1.mk*) were suppressed under both treatments, whereas *PavAP2/EREBP28* (*Pav_sc0001900.1_g290.1.mk*) was activated under both treatments. Moreover, all three DOF transcription factors (*PavDOF1.4-like*, *PavDOF1.5*, and *PavDOF3.4*) were upregulated under both treatments. Similarly, two identified HD-ZIP transcription factors (*PavHAT5* and *PavHOX3-like*) were activated under both treatments.

### DEGs related to waterlogging stress proteins

**Fig. 11:**
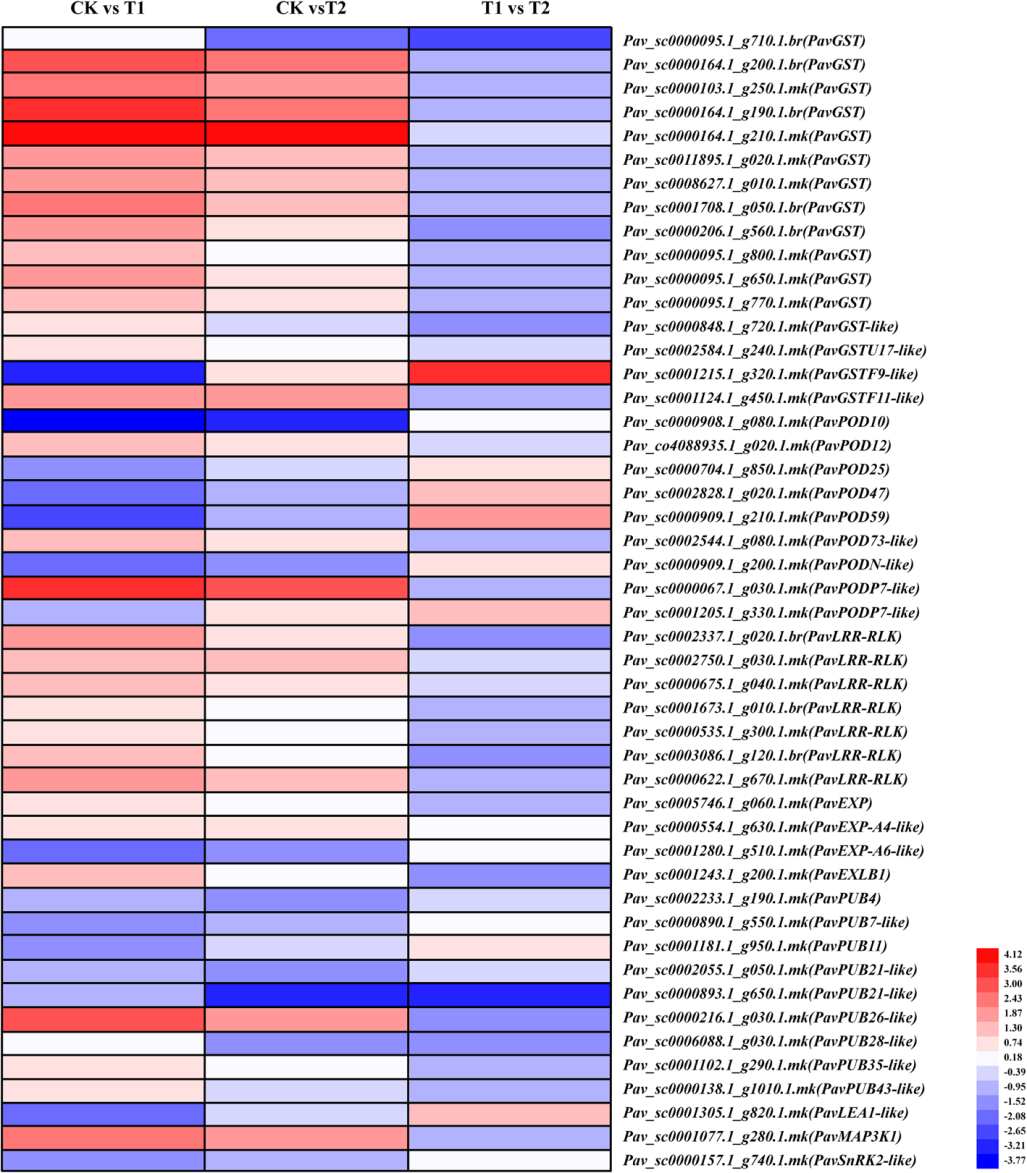
Expression profiles of differentially expressed genes related to stress associated genes, represented using a heatmap. The scale of color intensity is in the lower left quarter of heat map, representing the log_2_Fold-change values. Fold-change refers to the ratio of gene expression level in cherry rootstock leaves between control (CK) and treatments (T1/T2)

Proteins induced by exposure to excessive water have been recognized and proven to play important roles in waterlogging tolerance in various species (Pelah et al. 1997). Thus, in addition to the abovementioned DEGs, genes encoding several stress-related proteins, such as glutathione S-transferase (GST), peroxidase (POD), leucine-rich repeat receptor-like protein (LRR-RLK), late embryogenesis abundant protein (LEA), and mitogen-activated protein kinase kinase kinase (MAP3K), were also identified in our RNA-seq analysis (Fig. 11 and Table S16). Sixteen DEGs (including *PavGSTs*, *PavGST-like*, *PavGSTU17-like*, *PavGSTF9-like*, and *PavGSTF11-like*) encoding GSTs were identified for further analyses. Most of these genes were significantly upregulated under T1, and only *PavGSTF9-like* was suppressed. Under T2, the upregulation of *PavGSTs* was more moderate, indicating that plantlets under the T2 treatment were not as stressed as those under T1. Three transcripts of *PavPODs*, *PavPOD12* (*Pav_co4088935.1_g020.1.mk*), *PavPOD73-like* (*Pav_sc0002544.1_g080.1.mk*), and *PavPODP7-like* (*Pav_sc0000067.1_g030.1.mk*) were remarkably induced under both treatments. However, other *PavPODs* were downregulated under both treatments, suggesting that various members may have different functions. All genes encoding LRR-RLKs were significantly activated under T1, whereas only three of the seven *PavLRR-RLKs* were significantly induced under T2. Mitogen-activated protein kinase (MAPK) can transmit stress signals; thus, MAP3K that activates MAPK plays an important role in plant response to stress. Here, *PavMAP3K1* (*Pav_sc0001077.1_g280.1.mk*) was remarkably upregulated under both treatments, suggesting that MAP3K may be sensitive to stress.

### Validation of RNA-seq data by qRT-PCR

**Fig. 12:**
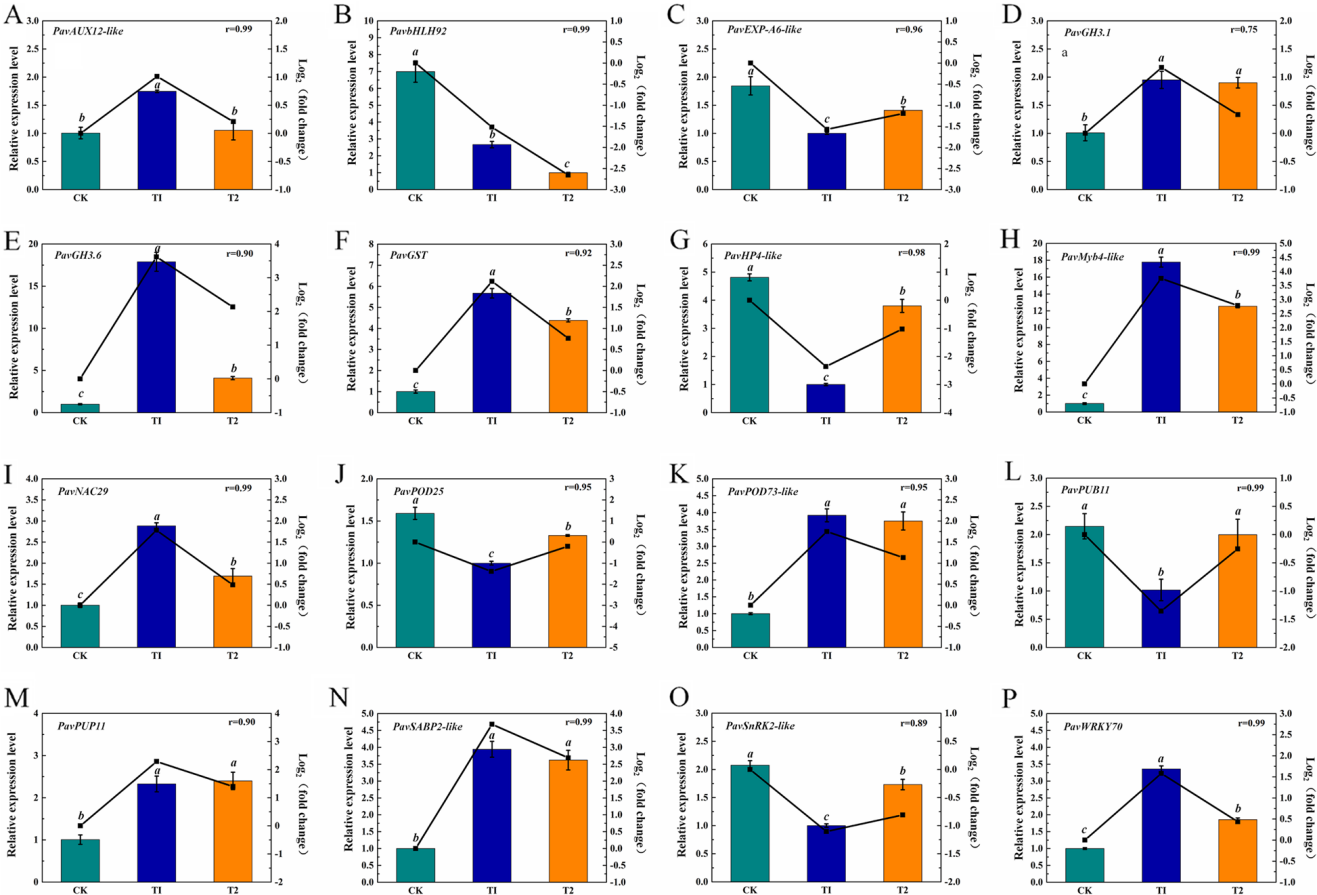
Quantitative reverse-transcription PCR (qRT-PCR) validation of gene expression levels of 16 selected DEGs related to hormone pathways and stress associated transcription factors and genes. The correlation coefficient was shown in the top right corner. The data are represented as mean ± standard error (SE) of three replicates. Statistical significance was determined using a one-way ANOVA; significant differences among means (LSD, *p* < 0.05) are indicated by different lowercase letters

To verify the accuracy and reliability of the RNA-seq data, the transcriptional levels of the 16 DEGs were analyzed by real-time quantitative PCR. These DEGs included IAA-related genes *PavAUX12-like* (*Pav_sc0002233.1_g180.1.mk*), *PavGH3.1* (*Pav_sc0001422.1_g120.1.mk*) and *PavGH3.6* (*Pav_sc0000269.1_g440.1.mk*), CTK-related genes *PavPUP11* (*Pav_sc0001243.1_g270.1.mk*) and *PavHP4-like* (*Pav_sc0000800.1_g510.1.mk*), SA-related gene *PavSABP2-like* (*Pav_sc0000348.1_g400.1.mk*), stress-related proteins *PavGST* (*Pav_sc0000206.1_g560.1.br*), *PavPOD25* (*Pav_sc0000704.1_g850.1.mk*), *PavPOD73-like* (*Pav_sc0002544.1_g080.1.mk*), *PavPUB11* (*Pav_sc0001181.1_g950.1.mk*), *PavSnRK2-like* (*Pav_sc0000157.1_g740.1.mk*) and *PavEXP-A6-like* (*Pav_sc0001280.1_g510.1.mk*), and transcription factors *PavbHLH92* (*Pav_sc0000998.1_g640.1.mk*), *PavMyb4-like* (*Pav_sc0000119.1_g370.1.mk*), *PavNAC29* (*Pav_sc0000244.1_g070.1.mk*) and *PavWRKY70* (*Pav_sc0001582.1_g320.1.mk*). The correlation between RNA-seq and qRT-PCR was shown in Fig. 12. The correlation coefficients were calculated, indicating the reliability of the data.

## Discussion

### Morphological, physiological and antioxidant properties of cherry rootstocks under waterlogging with or without oxygenation

The threat posed by waterlogging to crop yield is increasing, warranting the exploration of plant adaptation mechanisms to devise strategies to enhance plant waterlogging tolerance. In China, over half of the sweet cherry production regions have more precipitation and heavy or extreme rainfall days in the last 40 years (Fig. 2), which have likely caused flooding conditions and thus would potentially affect yields and fruit qualities of sweet cherry. Therefore, the waterlogging threat facing the sweet cherry industry must be evaluated. Waterlogging adversely affects plant growth and development, resulting in the closure of leaf stomata, chlorophyll degradation, leaf yellowing, senescence, and peeling, thereby reducing the photosynthetic capacity of plants (Kuai et al. 2014; Yan et al., 2018). Decreases in the chlorophyll content and photosynthetic rate have been reported in several species, including sesame, maize, and alfalfa (Zeng et al. 2019). In this study, cherry rootstocks also exhibited degradation of photosynthetic pigments, and consequently, a decrease in photosynthetic capacity (Fig. 3C-D), which was consistent with previous studies.

Reactive oxygen species (ROS) are metabolic products of plants that accumulate in response to stress. Although ROS can serve as signaling molecules in stress-related reactions, excessive ROS levels can severely damage proteins, DNA, cell membranes, and organelles (Baxter et al. 2014). Despite the requirement of oxygen for ROS production, these moieties accumulate in flooded environments (Pucciariello et al. 2012). Plants use antioxidant enzymes to maintain the dynamic balance of ROS levels and reduce oxidative damage (Hasanuzzaman et al. 2020). Waterlogging stress leads to enhancedactivities of SOD, CAT, and antioxidants, such as ascorbic acid and glutathione (Steffens et al. 2013). Furthermore, the activities of these enzymes vary in different plant lines, with waterlogging-resistant lines exhibiting higher enzyme activities than susceptible lines (Bansal and Srivastava 2012). In addition to genomic differences, the application of exogenous regulatory compounds also affects the activities of antioxidant enzymes. Salah et al. (2019) reported that γ-aminobutyric acid application enhances the activities of SOD, CAT, and APX. At low concentrations, H_2_O_2_ triggers the antioxidant system in soybeans and suppresses the production of ROS, reducing the consequent damage and promoting flooding tolerance (Andrade et al. 2018). In accordance with previous studies, in our study, SOD and CAT activities also increased after waterlogging, and seedlings under waterlogging conditions without oxygenation showed a more prominent increase in these activities (Fig. 5B-C). Therefore, oxygenation in our treatments may function similarly to exogenous regulatory compounds, such as γ-aminobutyric acid and H_2_O_2_, which can enhance the waterlogging tolerance of plants.

### Energy production under waterlogging conditions, with and without oxygenation

Hypoxia caused by waterlogging leads to an energy deficit in plants. The oxygen diffusion rates, in water and air, are very different, being 10,000 times slower in water than in air. As oxygen is the terminal electron acceptor in the mitochondrial electron transport chain, its deficiency inhibits mitochondrial respiration and decreases ATP production efficiency (Bailey-Serres and Voesenek 2008). Under low-oxygen conditions, plants obtain energy through glycolysis and ethanol fermentation. Although 1 mol glucose produces 36 to 38 mol ATP through the aerobic tricarboxylic acid cycle, only 2 mol ATP is produced through glycolysis and ethanol fermentation (Baxter-Burrell et al. 2002).

Several DEGs involved in glycolysis and ethanol fermentation (*ADH* and *PDC*) were remarkably accumulated in waterlogging-tolerant and waterlogging-sensitive cucumber lines after 2 d of waterlogging (Xu et al. 2017). Qi et al. (2014) reported that 14 genes encoding enzymes of the glycolysis/gluconeogenesis pathway and two genes encoding ADH and PDC in Taxodium ‘Zhongshansa’ roots were deferentially expressed in response to 1 h of waterlogging, 14 being upregulated and two (encoding GAPDH) being downregulated. Similarly, in our study, three critical genes involved in glycolysis, *Pav6PGD3*, *PavPFK5*, and *PavENO1*, and two (*PavADH-like 4*, and *PavADH6*) of the three genes encoding ADH were significantly upregulated under waterlogging conditions, whereas *PavADH* was suppressed, which is slightly different from previous studies. This difference may result from the fact that most researchers selected roots as their samples, whereas we used leaves.

Sucrose synthase is crucial for supplying sugar to plants under abiotic stress; thus, the regulation of this enzyme influences plant stress tolerance. Maize *sucrose synthase* mutants are more sensitive to low-oxygen conditions than the wild-type, whereas the overexpression of *sucrose synthase* (*SuSy*) enhances tolerance to low-oxygen conditions in cucumber (Ricard et al. 1998; Wang et al., 2014). Xu et al. (2017) reported that under waterlogging, the gene encoding sucrose synthase was significantly induced in waterlogging-tolerant cucumber line ‘Zaoer-N’, but no obvious change was observed in the waterlogging-sensitive ‘Pepino’. In our waterlogging treatments, *PavSuSy1* was highly activated, suggesting that *sucrose synthase* may also be important for waterlogging tolerance in cherry rootstocks.

### Phytohormones under waterlogging conditions, with and without oxygenation

Phytohormones play critical roles in regulating the plant lifecycle, and a dynamic balance of phytohormones is fundamental to plant growth and development (Bartoli et al. 2013; Wang et al., 2020). Furthermore, plant hormones integrate various signal transduction pathways to adapt to abiotic stress (Wolters and Jürgens 2009).

Ethylene is a critical phytohormone in plant responses to flooding, and its biosynthesis is induced under waterlogging conditions (Hattori et al. 2009). 1-Aminocyclopropane-1-carboxylic acid (ACC) is produced by catalysis with ACC synthase (ACS) and is subsequently converted to ethylene by ACC oxidase (ACO). Therefore, both ACS and ACO are critical enzymes in ethylene biosynthesis. In *Arabidopsis*, waterlogging induces *ACS* and *ACO5*, thereby, increasing ethylene biosynthesis (Rauf et al. 2013). In rice roots, *ACS1* and *ACO5* are activated under hypoxia (Yamauchi et al. 2017). Similarly, in our study, all genes encoding ACS and ACO were upregulated under waterlogging conditions, with plants under T1 showing greater upregulation than that under T2 (Fig. 7). ERF is a transcription factor regulated by ethylene that functions in plant adaptation to various biotic and abiotic stresses. Several *ERFs* identified in our study were differentially expressed, indicating different functions of members of the ERF family in waterlogging tolerance.

ABA is an important phytohormone that resizes guard cells and regulates the stomata, thereby adjusting the water potential of plants (Zhu 2016; He et al. 2018). In *Solanum dulcamara*, waterlogging stress suppresses ABA biosynthesis and induces ABA degradation, resulting in a decrease in the ABA concentration in plant stems (Dawood et al. 2016). In soybean, a decrease in ABA concentration also occurred under waterlogging conditions at 5 and 10 DAT (Kim et al. 2015b). Likewise, in our study, *PavNCED5,* which catalyzes a rate-limiting reaction in ABA biosynthesis, was significantly downregulated after waterlogging, indicating suppression of ABA biosynthesis. In contrast, *PavABAox2*, *PavABAox4*, and *PavABAox4-like*, which are involved in ABA degradation, were repressed. This may be because the selected samples were leaves, considering that in studies on cotton and wheat, ABA accumulation was also observed in the aboveground parts under waterlogging conditions (Zhang et al. 2016; Nan et al. 2002).

Other phytohormones, such as IAA, GA, and SA, also play important roles in plant adaptation to waterlogging stress. IAA is involved in the formation of adventitious roots (ARs), which are critical for plant waterlogging tolerance (Agulló-Antón et al. 2014; Qi et al. 2019). *PIN2*, a polar auxin transporter, was activated in *Solanum dulcamara* under flooding stress, and its deactivation repressed the initiation of AR primordium (Dawood et al. 2016). Here, *PavPIN1b* was also induced by waterlogging (Fig. 9). Furthermore, in *Arabidopsis*, three auxin-induced *Gretchen Hagen3* (*GH3*) genes, *GH3.3*, *GH3.5*, and *GH3.6* involved in acyl-acid-amido biosynthesis were reported to be essential for ARs formation (Gutierrez et al. 2012). In this study, *PavGH3.1* and *PavGH3.6* were induced under waterlogging conditions, whereas *PavGH3.5* was suppressed, suggesting that these genes may function differently in different species.

GA functions in plant responses to flooding stress (Kuroha et al. 2018; Nagai et al. 2020). Treatment with inhibitors or mutations in GA biosynthesis and signal transduction genes suppresses internode elongation under waterlogging conditions (Ayano et al. 2014). In our study, several important genes, such as *PavGA2ox1-like*, *PavGA2ox8*, and *PavGA2ox8-like*, were activated after the waterlogging treatments, but *PavGA3ox* was suppressed (Figure S8).

SA can activate stress-related genes to improve plant adaptability to various environments (Arif et al., 2020; Hayat et al. 2010). In soybeans subjected to waterlogging, the SA content in tolerant lines increased remarkably at 5 and 10 DAT, whereas there was no significant change in sensitive lines (Kim et al. 2015b). In waterlogged peaches, treatment with exogenous SA enhanced various physiological parameters, such as ethanol dehydrogenase, POD, and CAT activities, thereby, improving their adaptability to waterlogging stress (Wang et al., 2015). In the present study, two genes involved in SA signal transduction, *PavSABP2-like* (*Pav_sc0000348.1_g400.1.mk* and *Pav_sc0000348.1_g500.1.mk*), were significantly activated under waterlogging conditions, which is consistent with the results of previous studies.

## Methods

### Plant materials and treatment

Three-month-old cherry rootstocks ‘Y1’, ‘Colt’, ‘Gisela 5’ (‘G5’), ‘Gisela 6’ (‘G6’), ‘Gisela 12’ (‘G12’), were grown in pots (8 cm diameter) containing garden soil, matrix and vermiculite (2:2:1, v/v/v) at 23 °C under a 16:8 h light: dark (L:D) cycle at the Shanghai Jiao Tong University, Shanghai, China (31°11N, 121°29W). Before treatment, all rootstocks were regularly irrigated and randomly subjected to three treatments: normally- watered (control; CK), completely immersed in water, and the water surface 1 cm above the soil surface (T1), and completely immersed in water, but oxygenated using an oxygen pump (T2). The water level was maintained throughout the experiment. To evaluate the waterlogging resistance of different rootstocks, samples were collected at 0, 0.25, 0.5, 1.5, 3, 6, and 12 days after treatment (DAT), frozen in liquid nitrogen, and stored at –80 °C. For analysis of cherry rootstock response to waterlogging, only ‘G6’ samples were collected on 0, 0.25, 0.5, 1, 2, 4, 8, and 16 DAT, frozen in liquid nitrogen and stored at –80 °C. Three biological replicates were used for each sample in both experiments.

### Study area and climate scenarios

The study regions included 15 stations that produce sweet cherry, which located in Hefei (31.78°N, 117.30°E), Tianshui (34.57°N, 105.74°E), Guiyang (26.59°N, 106.73°E), Qinhuangdao (39.85°N, 119.52°E), Shijiazhuang (38.07°N, 114.35°E), Zhengzhou (34.71°N, 113.66°E), Dalian (38.91°N, 121.64°E), Yingkou (40.67°N, 122.17°E), Linqu (36.47°N, 118.56°E), Fushan (37.48°N, 121.23°E, a district of Yantai city), Chengcheng (35.18°N, 109.92°E), Tongchuan (35.08°N, 109.07°E), Minhang (31.10°N, 121.37°E, a district of Shanghai city), Wenjiang (30.75°N, 103.86°E, a district of Chengdu city), and Kashi (39.49°N, 75.75°E). Among them, Fushan, Linqu, Chengcheng, Tongchuan, Yingkou, Dalian, Zhengzhou, Wenjiang, Shijiazhuang, Qinhuangdao and Tianshui stations locate in the main sweet cherry production areas, and Hefei, Guiyang, Minhang and Kashi represent emerging sweet cherry regions. The meteorological dataset observed from 1982 to 2021 was obtained from the National Meteorological Information Center of China (http://data.cma.cn/).

### Measurement of relative water content

Relative water content (RWC) was measured using the fresh weight method. Fresh leaves were gently washed and wiped with absorbent paper, and their fresh weight (FW) was determined. They were immersed in distilled water for 8 h and wiped again to determine turgid weight (TW). Finally, the leaves were dried at 60 °C for 24 h to determine the dry weight (DW). RWC (%) was calculated using the following formula: RWC (%) = (FW–DW)/(TW–DW) × 100. Five replicates were performed for each treatment.

### Measurement of chlorophyll fluorescence

The chlorophyll fluorescence parameter (Fv/Fm) was measured using an FMS-2 Pulse Modulated Fluorometer (Hansatech, England). The sample was kept in the dark for 30 min and the initial fluorescence (F_0_) was measured. The maximum fluorescence (Fm) was measured after exposure to saturated pulsed light (5000 μmol m^−2^·s^−1^) for 0.7 s. Fv/Fm was calculated as follows: Fv/Fm = (Fm–F_0_)/Fm. Five replicates were performed for each treatment.

### Measurement of photosynthetic parameters

Photosynthetic parameters were determined using a portable photosynthesis system (LI-6800, United States) at an air flow speed of 500 μmol·s^−1^ and photosynthetically active radiation (PAR) of 1000 μmol·m^−2^·s^−1^. The CO_2_ concentration in the leaf chamber was maintained at 400 μmol·mol^−1^ using CO_2_ cylinders. These parameters were recorded in five replicates.

### Measurement of antioxidant enzyme activities

Superoxide dismutase (SOD, BC0170) and catalase (CAT, BC0200) activities were measured using commercial kits (Solarbio Life Sciences, Beijing, China) according to the manufacturer’s protocol.

### RNA extraction, cDNA library preparation, and Illumina sequencing

Total RNA was extracted from the leaf samples using a plant RNA purification kit (TIANGEN Biotech, Beijing, China) and treated with RNase-free DNase I (Takara, Japan) to prevent genomic DNA contamination. The RNA integrity was confirmed using an Agilent 2100 Bioanalyzer (Agilent Technologies, Palo Alto, CA, USA). RNA quality was measured using agarose gel electrophoresis (Jiu et al. 2019, 2020). The RNA quality parameters were as follows: RNA integrality numbers ≥ 7.5, A260/A280 between 1.9 and 2.10, and 28S:18S RNA ratio > 1. For Illumina sequencing, poly (A)^+^ RNA was separated from total RNA using Dynal oligo (dT)25 beads, according to the manufacturer’s protocol. The cDNA libraries of the cherry rootstock leaves were constructed as described by Quan et al. (2017) and sequenced on a NovaSeq platform (Illumina) by Shanghai Personal Biotechnology Co. Ltd.

### Read mapping and differential expression analysis

Raw reads obtained from the NovaSeq sequencing platform were processed to remove shorter reads, adapter sequences, and low-quality reads. The clean reads were aligned to the reference *P. avium* genome (https://www.rosaceae.org/species/prunus_avium/genome_v1.0.a1) using HISAT2 (http://ccb.jhu.edu/software/hisat2/index.shtml) (Kim et al. 2015a). Uniquely mapped reads were used for further analyses. Normalized expression levels were determined using fragments per kilobase of exon per million fragments mapped (FPKM), based on the number of uniquely mapped reads, to remove the impact of different gene lengths and sequencing differences on the calculation of gene expression. Differential expression analysis between the treatments and control were performed using the ‘DESeq’ R package (Anders and Huber, 2010). The reliability of differential transcript accumulation was evaluated using the *p*-value (Audic and Claverie 1997). The criteria for classification were significantly differentially expressed genes (DEGs) with *p* < 0.05, and genes with a minimum of two-fold difference in expression (|log_2_Fold change|> 1). Volcano and MA maps of the DEGs were generated using the ‘gplots2’ package in R. The ‘heatmap’ R package was used to perform two-way clustering analysis of DEGs in all comparison groups using Euclidean distance and complete linkage.

### Gene annotation and enrichment analysis

All DEGs were annotated based on a BLAST search and searched against protein databases, such as NCBI non-redundant sequence (NCBI Nr), Clusters of Orthologous Groups (COGs), Swiss Institute of Bioinformatics databases (Swiss-Prot), and KEGG. All genes were mapped to terms in the gene ontology (GO) database, and the number of DEGs was calculated for each term. We then used topGO to perform GO enrichment analysis on DEGs, calculated *p*-value using the hypergeometric distribution method (the standard of significant enrichment is *p* < 0.05) and found the GO terms associated with significantly enriched differential genes to determine their main biological functions (Zhang et al. 2022). The statistical enrichment of DEGs was tested using the ClusterProfiler (version 3.4.4) software, and *p* < 0.05 was considered significantly enriched in KEGG pathways (Ju et al. 2019).

### Verification of gene expression by qRT-PCR

To confirm the quality of the RNA-seq data, 16 DEGs were randomly selected for qRT-PCR analysis using a CFX Connect Real-Time System. The PCR mixture (10 µL) included 1 µL cDNA template, 5 µL of 2 × TB Green II mix, and 0.5 µL each of reverse and forward primer. The cycling parameters are as described by Jia et al. (2016). For each treatment, the samples were from three plantlets with uniform growth. The 2^–ΔΔCT^ method was used to compute the relative expression level of each tested gene (Livak and Schmittgen 2001). *PavActin* was used as an internal reference control. The primers used for qRT-PCR are listed in Table S1.

### Statistical analysis

A completely randomized design (CRD) was used in the experiment, which included three biological replicates. The data were analyzed using SAS software (SAS Institute). Statistical differences were determined using a one-way ANOVA at a significance level of *p* < 0.05. Data are presented as the mean ± standard deviation (SD) of more than three replicates.

### Supplementary Information

**Additional file 1: Fig. S1.** Phenotypic traits of aboveground and underground parts of five cherry rootstocks under waterlogging conditions with or without oxygenation. Bar = 5 cm. **Fig. S2.** Overview of the transcriptomes of cherry rootstock leaves under control (CK) and waterlogging conditions with (T2) or without oxygenation (T1). Pairwise correlation of biological replicates from CK, T1, and T2. **Fig. S3.** Circular visualization of the genomic alterations in cherry rootstock (*Cerasus* spp.) under CK, T1, and T2. Red and green histograms represent the log_2_fold-change values for up- and downregulated genes, respectively. The gray scatter plot shows the log_2_fold-change values for the non-differentially expressed genes. **Fig. S4.** Transcriptional changes in cherry rootstock (*Cerasus* spp.) leaves after 8 d under CK, T1, and T2. (A) Expression profiles of genes following different treatments as indicated are represented using the heatmap. (B) The number of up- and downregulated genes in different treatments (C) Venn diagrams show the proportions of differentially expressed genes (DEGs) in three comparisons. CK, control; T1, waterlogging stress, T2, waterlogging stress with oxygenation. Significance analysis for the DEGs in CK *vs*. T1 (D), CK *vs*. T2 (E)and T1 *vs*. T2 (F) comparisons using volcano plots. **Fig. S5.** Gene ontology classification and enrichment analysis of the differentially expressed genes in CK *vs*. T1, CK *vs*. T2, and T1 *vs*. T2 comparisons. **Fig. S6.** KEGG pathway classification (A) and enrichment analysis (B) of the differentially expressed genes in CK *vs*. T1, CK *vs*. T2, and T1 *vs*. T2 comparisons. **Fig. S7.** Expression profiles of differentially expressed genes related to CTK biosynthesis, transport and signaling pathways, represented using a heatmap. The scale of color intensity is shown in the lower left quarter of heatmap, representing the log_2_fold-change values. Fold-change refers to the ratio of gene expression levels in cherry rootstock leaves between control (CK) and treatments (T1/T2). **Fig. S8.** Expression profiles of differentially expressed genes related to gibberellin (GA), brassinosteroids (BR), and salicylic acid (SA) biosynthesis and signaling pathways, shown using a heatmap. The scale of color intensity is shown in the lower left quarter of heat map representing the log_2_Fold-change values. Fold-change refers to the ratio of gene expression levels in cherry rootstock leaves between control (CK) and treatments (T1/T2).

**Additional file 2: Table S1.** Sequence of primers used for quantitative reverse-transcription PCR. **Table S2.** Summary of the sequence data analysis. **Table S3.** Summary of RNA-Seq map. **Table S4.** KEGG pathway enrichment of differentially expressed genes in T1 *vs*. CK comparison. **Table S5.** KEGG pathway enrichment of differentially expressed genes in T2 *vs*. CK comparison. **Table S6.** KEGG pathway enrichment of differentially expressed genes in T2 *vs*. T1 comparison. **Table S7.** Expression profiles of differentially expressed genes associated with energy production. **Table S8.** Expression profiles of differentially expressed genes in the ethylene metabolic pathway. **Table S9.** Expression profiles of differentially expressed genes in the abscisic acid metabolic pathway. **Table S10.** Expression profiles of differentially expressed genes in the cytokinin metabolic pathway. **Table S11.** Expression profiles of differentially expressed genes in the auxin metabolic pathway. **Table S12.** Expression profiles of differentially expressed genes in the gibberellin metabolic pathway. **Table S13.** Expression profiles of differentially expressed genes in the salicylic acid metabolic pathway. **Table S14.** Expression profiles of differentially expressed genes in the brassinosteroid metabolic pathway. **Table S15.** Expression profiles of differentially expressed genes related to stress-associated transcription factors. **Table S16.** Expression profiles of differentially expressed genes related to stress.

## Data Availability

All data generated or analyzed during this study are included in this published article.

## Abbreviations

DEGs: Differentially Expressed Genes
SRA: Sequence Read Archive
RWC: Relative Water Content
Pn: Net Photosynthetic Rate
BP: Biological Process
MF: Molecular Function
CC: Cellular Component
NRTs: Nitrate Transporters
nsHBs: Non-symbiotic Hemoglobins
ETH: Ethylene
ABA: Abscisic Acid
IAA: Indole-3-acetic Acid
CTK: Cytokinin
GA: Gibberellin
BR: Brassinosteroids
SA: Salicylic Acid
GST: Glutathione S-transferase
POD: Peroxidase
LRR-RLK: Leucine-rich Repeat Receptor-like Protein
LEA: Late Embryogenesis Abundant Protein
MAP3K: Mitogen-activated Protein Kinase Kinase Kinase
MAPK: Mitogen-activated Protein Kinase
ROS: Reactive Oxygen Species
SuSy: Sucrose Synthase
ACC: 1-Aminocyclopropane-1-carboxylic Acid
ACS: ACC Synthase
ACO: ACC Oxidase
AR: Adventitious Root
G5: Gisela 5
G6: Gisela 6
G12: Gisela 12
DAT: Days After Treatment
FW: Fresh Weight
TW: Turgid Weight
Fv/Fm: The Chlorophyll Fluorescence Parameter
F_0_: The Initial Fluorescence
Fm: The Maximum Fluorescence
PAR: Photosynthetically Active Radiation
FPKM: Fragments Per Kilobase of Exon Per Million Fragments Mapped
COGs: Clusters of Orthologous Groups
Swiss-Prot: Swiss Institute of Bioinformatics databases
GO: Gene Ontology
CRD: Completely Randomized Design
SD: Standard Deviation
