# Supplementary figures and images for: Oxygenation alleviates waterlogging-caused damages to cherry rootstocks
Source: Mol Hortic. 2023 Apr 17;3:8. doi: 10.1186/s43897-023-00056-1 (PMC10515082; doi:10.1186/s43897-023-00056-1)

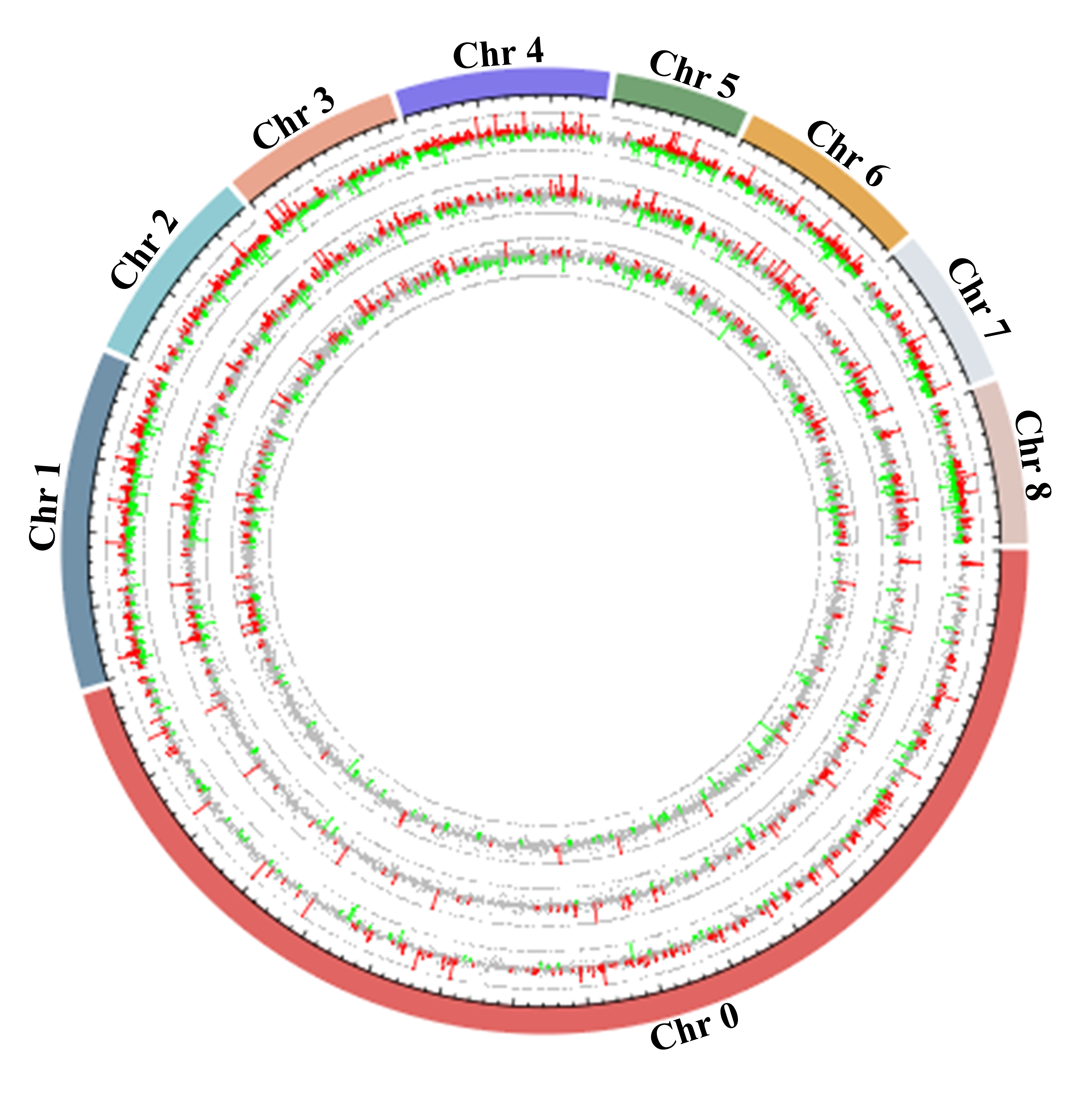

Supplement: Supplementary file 1 — Additional file 1: Fig. S1. Phenotypic traits of aboveground and underground parts of five cherry rootstocks under waterlogging conditions with or without oxygenation. Bar = 5 cm. Fig. S2. Overview of the transcriptomes of cherry rootstock leaves under control (CK) and waterlogging conditions with (T2) or without oxygenation (T1). Pairwise correlation of biological replicates from CK, T1, and T2. Fig. S3. Circular visualization of the genomic alterations in cherry rootstock (Cerasus spp.) under CK, T1, and T2. Red and green histograms represent the log2fold-change values for up- and downregulated genes, respectively. The gray scatter plot shows the log2fold-change values for the non-differentially expressed genes. Fig. S4. Transcriptional changes in cherry rootstock (Cerasus spp.) leaves after 8 d under CK, T1, and T2. (A) Expression profiles of genes following different treatments as indicated are represented using the heatmap. (B) The number of up- and downregulated genes in different treatments (C) Venn diagrams show the proportions of differentially expressed genes (DEGs) in three comparisons. CK, control; T1, waterlogging stress, T2, waterlogging stress with oxygenation. Significance analysis for the DEGs in CK vs. T1 (D), CK vs. T2 (E)and T1 vs. T2 (F) comparisons using volcano plots. Fig. S5. Gene ontology classification and enrichment analysis of the differentially expressed genes in CK vs. T1, CK vs. T2, and T1 vs. T2 comparisons. Fig. S6. KEGG pathway classification (A) and enrichment analysis (B) of the differentially expressed genes in CK vs. T1, CK vs. T2, and T1 vs. T2 comparisons. Fig. S7. Expression profiles of differentially expressed genes related to CTK biosynthesis, transport and signaling pathways, represented using a heatmap. The scale of color intensity is shown in the lower left quarter of heatmap, representing the log2fold-change values. Fold-change refers to the ratio of gene expression levels in cherry rootstock leaves between control (CK) [file 43897_2023_56_MOESM1_ESM.zip › Figure S1-S8/Figure S3.tif]

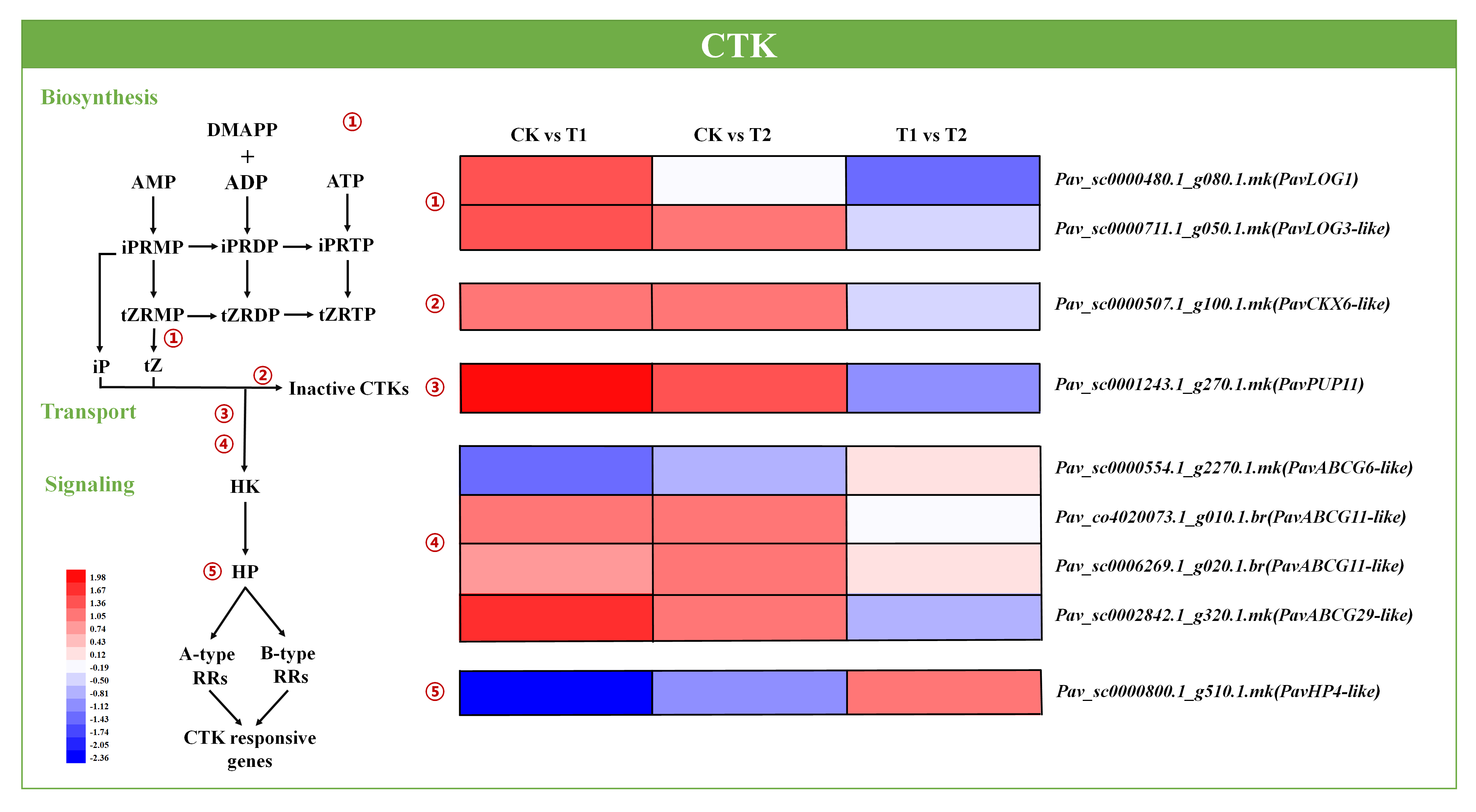

Supplement: Supplementary file 1 — Additional file 1: Fig. S1. Phenotypic traits of aboveground and underground parts of five cherry rootstocks under waterlogging conditions with or without oxygenation. Bar = 5 cm. Fig. S2. Overview of the transcriptomes of cherry rootstock leaves under control (CK) and waterlogging conditions with (T2) or without oxygenation (T1). Pairwise correlation of biological replicates from CK, T1, and T2. Fig. S3. Circular visualization of the genomic alterations in cherry rootstock (Cerasus spp.) under CK, T1, and T2. Red and green histograms represent the log2fold-change values for up- and downregulated genes, respectively. The gray scatter plot shows the log2fold-change values for the non-differentially expressed genes. Fig. S4. Transcriptional changes in cherry rootstock (Cerasus spp.) leaves after 8 d under CK, T1, and T2. (A) Expression profiles of genes following different treatments as indicated are represented using the heatmap. (B) The number of up- and downregulated genes in different treatments (C) Venn diagrams show the proportions of differentially expressed genes (DEGs) in three comparisons. CK, control; T1, waterlogging stress, T2, waterlogging stress with oxygenation. Significance analysis for the DEGs in CK vs. T1 (D), CK vs. T2 (E)and T1 vs. T2 (F) comparisons using volcano plots. Fig. S5. Gene ontology classification and enrichment analysis of the differentially expressed genes in CK vs. T1, CK vs. T2, and T1 vs. T2 comparisons. Fig. S6. KEGG pathway classification (A) and enrichment analysis (B) of the differentially expressed genes in CK vs. T1, CK vs. T2, and T1 vs. T2 comparisons. Fig. S7. Expression profiles of differentially expressed genes related to CTK biosynthesis, transport and signaling pathways, represented using a heatmap. The scale of color intensity is shown in the lower left quarter of heatmap, representing the log2fold-change values. Fold-change refers to the ratio of gene expression levels in cherry rootstock leaves between control (CK) [file 43897_2023_56_MOESM1_ESM.zip › Figure S1-S8/Figure S7.tif]

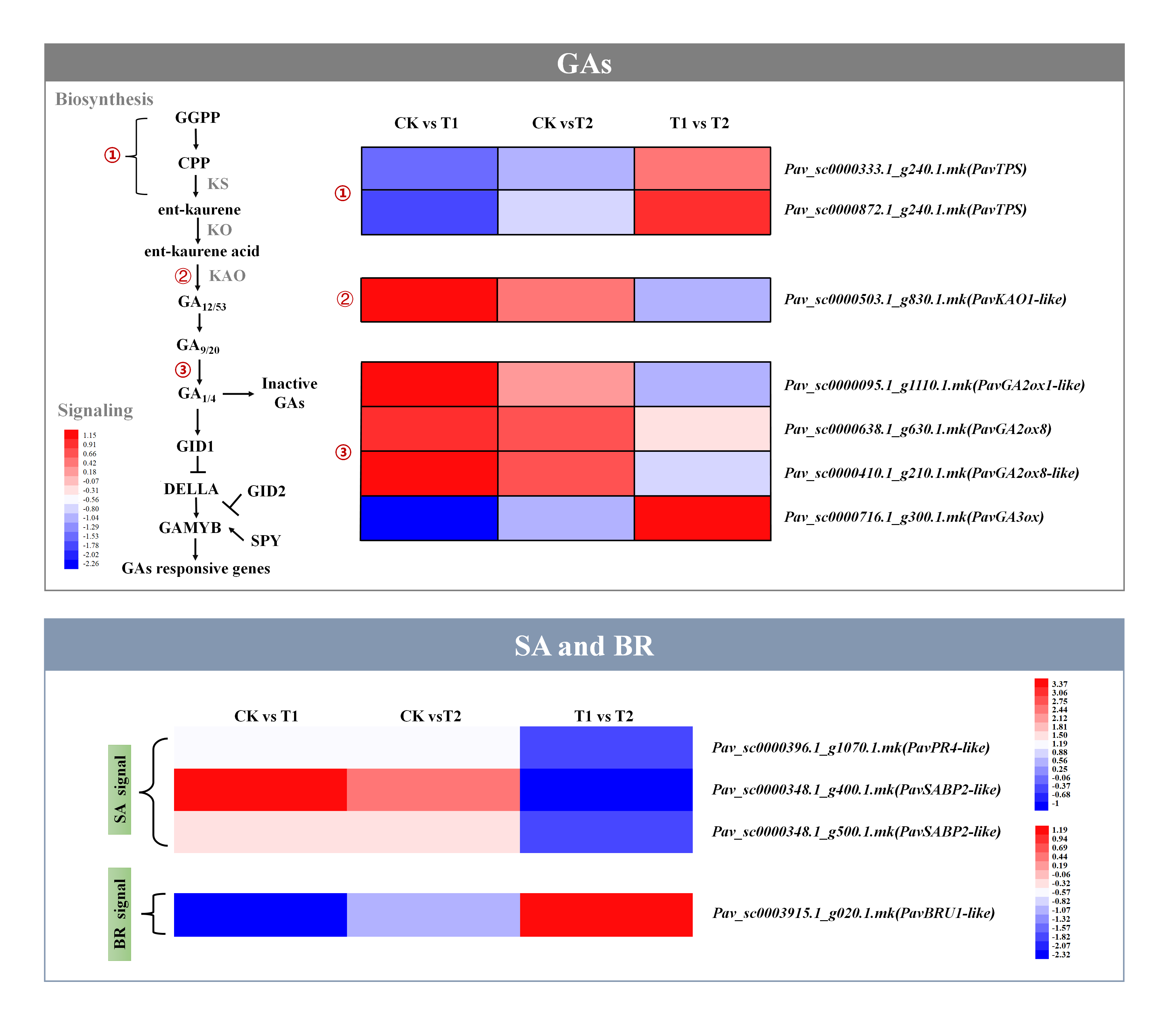

Supplement: Supplementary file 1 — Additional file 1: Fig. S1. Phenotypic traits of aboveground and underground parts of five cherry rootstocks under waterlogging conditions with or without oxygenation. Bar = 5 cm. Fig. S2. Overview of the transcriptomes of cherry rootstock leaves under control (CK) and waterlogging conditions with (T2) or without oxygenation (T1). Pairwise correlation of biological replicates from CK, T1, and T2. Fig. S3. Circular visualization of the genomic alterations in cherry rootstock (Cerasus spp.) under CK, T1, and T2. Red and green histograms represent the log2fold-change values for up- and downregulated genes, respectively. The gray scatter plot shows the log2fold-change values for the non-differentially expressed genes. Fig. S4. Transcriptional changes in cherry rootstock (Cerasus spp.) leaves after 8 d under CK, T1, and T2. (A) Expression profiles of genes following different treatments as indicated are represented using the heatmap. (B) The number of up- and downregulated genes in different treatments (C) Venn diagrams show the proportions of differentially expressed genes (DEGs) in three comparisons. CK, control; T1, waterlogging stress, T2, waterlogging stress with oxygenation. Significance analysis for the DEGs in CK vs. T1 (D), CK vs. T2 (E)and T1 vs. T2 (F) comparisons using volcano plots. Fig. S5. Gene ontology classification and enrichment analysis of the differentially expressed genes in CK vs. T1, CK vs. T2, and T1 vs. T2 comparisons. Fig. S6. KEGG pathway classification (A) and enrichment analysis (B) of the differentially expressed genes in CK vs. T1, CK vs. T2, and T1 vs. T2 comparisons. Fig. S7. Expression profiles of differentially expressed genes related to CTK biosynthesis, transport and signaling pathways, represented using a heatmap. The scale of color intensity is shown in the lower left quarter of heatmap, representing the log2fold-change values. Fold-change refers to the ratio of gene expression levels in cherry rootstock leaves between control (CK) [file 43897_2023_56_MOESM1_ESM.zip › Figure S1-S8/Figure S8.tif]
