# Supplementary material for: Oxygenation alleviates waterlogging-caused damages to cherry rootstocks
Source: Mol Hortic. 2023 Apr 17;3:8. doi: 10.1186/s43897-023-00056-1 (PMC10515082; doi:10.1186/s43897-023-00056-1)
Supplement: Supplementary file 2 — Additional file 2: Table S1. Sequence of primers used for quantitative reverse-transcription PCR. Table S2. Summary of the sequence data analysis. Table S3. Summary of RNA-Seq map. Table S4. KEGG pathway enrichment of differentially expressed genes in T1 vs. CK comparison. Table S5. KEGG pathway enrichment of differentially expressed genes in T2 vs. CK comparison. Table S6. KEGG pathway enrichment of differentially expressed genes in T2 vs. T1 comparison. Table S7. Expression profiles of differentially expressed genes associated with energy production. Table S8. Expression profiles of differentially expressed genes in the ethylene metabolic pathway. Table S9. Expression profiles of differentially expressed genes in the abscisic acid metabolic pathway. Table S10. Expression profiles of differentially expressed genes in the cytokinin metabolic pathway. Table S11. Expression profiles of differentially expressed genes in the auxin metabolic pathway. Table S12. Expression profiles of differentially expressed genes in the gibberellin metabolic pathway. Table S13. Expression profiles of differentially expressed genes in the salicylic acid metabolic pathway. Table S14. Expression profiles of differentially expressed genes in the brassinosteroid metabolic pathway. Table S15. Expression profiles of differentially expressed genes related to stress-associated transcription factors. Table S16. Expression profiles of differentially expressed genes related to stress. [file 43897_2023_56_MOESM2_ESM.zip › Table S1-S16/Table S2.docx]

**Table S2** **Summary of the sequence data analysis.**

| Sample | Raw reads | Clean reads | Total bases (Gb) | Clean bases (Gb) | Q30 (Gb) | N (%) | Q20 (%) | Q30 (%) |
| --- | --- | --- | --- | --- | --- | --- | --- | --- |
| CK-1 | 50356264 | 43090762 | 7.55 | 6.46 | 6.84 | 0.001751 | 96.03 | 90.57 |
| CK-2 | 42955218 | 37811938 | 6.44 | 5.67 | 6.01 | 0.001749 | 97.4 | 93.21 |
| CK-3 | 44304198 | 37770164 | 6.65 | 5.67 | 6.20 | 0.00175 | 97.31 | 93.28 |
| T1-1 | 43779124 | 38750692 | 6.57 | 5.81 | 6.12 | 0.001794 | 97.41 | 93.21 |
| T1-2 | 42543972 | 37267070 | 6.38 | 5.59 | 5.94 | 0.001765 | 97.33 | 93.03 |
| T1-3 | 48676260 | 41889934 | 7.30 | 6.28 | 6.82 | 0.001742 | 97.42 | 93.46 |
| T2-1 | 41731796 | 37029532 | 6.26 | 5.55 | 5.84 | 0.001732 | 97.47 | 93.3 |
| T2-2 | 46194146 | 40551538 | 6.93 | 6.08 | 6.46 | 0.001767 | 97.41 | 93.24 |
| T2-3 | 60803764 | 45786676 | 9.12 | 6.87 | 8.51 | 0.001738 | 97.16 | 93.26 |

Note: Q30 (bp): The total number of bases with base recognition accuracy above 99.9%; N(%): Percentage of fuzzy bases; Q20(%): The percentage of bases with base recognition accuracy above 99%; Q30(%): The percentage of bases with base recognition accuracy above 99.9%。
