# Supplementary material for: Oxygenation alleviates waterlogging-caused damages to cherry rootstocks
Source: Mol Hortic. 2023 Apr 17;3:8. doi: 10.1186/s43897-023-00056-1 (PMC10515082; doi:10.1186/s43897-023-00056-1)
Supplement: Supplementary file 2 — Additional file 2: Table S1. Sequence of primers used for quantitative reverse-transcription PCR. Table S2. Summary of the sequence data analysis. Table S3. Summary of RNA-Seq map. Table S4. KEGG pathway enrichment of differentially expressed genes in T1 vs. CK comparison. Table S5. KEGG pathway enrichment of differentially expressed genes in T2 vs. CK comparison. Table S6. KEGG pathway enrichment of differentially expressed genes in T2 vs. T1 comparison. Table S7. Expression profiles of differentially expressed genes associated with energy production. Table S8. Expression profiles of differentially expressed genes in the ethylene metabolic pathway. Table S9. Expression profiles of differentially expressed genes in the abscisic acid metabolic pathway. Table S10. Expression profiles of differentially expressed genes in the cytokinin metabolic pathway. Table S11. Expression profiles of differentially expressed genes in the auxin metabolic pathway. Table S12. Expression profiles of differentially expressed genes in the gibberellin metabolic pathway. Table S13. Expression profiles of differentially expressed genes in the salicylic acid metabolic pathway. Table S14. Expression profiles of differentially expressed genes in the brassinosteroid metabolic pathway. Table S15. Expression profiles of differentially expressed genes related to stress-associated transcription factors. Table S16. Expression profiles of differentially expressed genes related to stress. [file 43897_2023_56_MOESM2_ESM.zip › Table S1-S16/Table S3.docx]

**Table S3** **Summary of RNA-Seq map.**

| Sample | Clean reads | Total_  Mapped (%) | Multiple_  Mapped (%) | Uniquely_  Mapped (%) | Map_Events | Mapped reads | Mapped_  to_Inter | Mapped_  to_Exon |
| --- | --- | --- | --- | --- | --- | --- | --- | --- |
| CK-1 | 43090762 | 36783338 (85.36%) | 1852304 (5.04%) | 34931034 (94.96%) | 34931034 | 30238827 (86.57%) | 4692207 (13.43%) | 28655373 (94.76%) |
| CK-2 | 37811938 | 33027896 (87.35%) | 1670222 (5.06%) | 31357674 (94.94%) | 31357674 | 27153349 (86.59%) | 4204325 (13.41%) | 25749474 (94.83%) |
| CK-3 | 37770164 | 32722672 (86.64%) | 1752772 (5.36%) | 30969900 (94.64%) | 30969900 | 26200442 (84.60%) | 4769458 (15.40%) | 25031117 (95.54%) |
| T1-1 | 38750692 | 33549110 (86.58%) | 1765106 (5.26%) | 31784004 (94.74%) | 31784004 | 27316604 (85.94%) | 4467400 (14.06%) | 25802656 (94.46%) |
| T1-2 | 37267070 | 32357114 (86.82%) | 1647243 (5.09%) | 30709871 (94.91%) | 30709871 | 26373849 (85.88%) | 4336022 (14.12%) | 24897999 (94.40%) |
| T1-3 | 41889934 | 36171820 (86.35%) | 1967957 (5.44%) | 34203863 (94.56%) | 34203863 | 28768603 (84.11%) | 5435260 (15.89%) | 27409928 (95.28%) |
| T2-1 | 37029532 | 32206396 (86.97%) | 1609094 (5.00%) | 30597302 (95.00%) | 30597302 | 26390163 (86.25%) | 4207139 (13.75%) | 24955936 (94.57%) |
| T2-2 | 40551538 | 35271466 (86.98%) | 1770994 (5.02%) | 33500472 (94.98%) | 33500472 | 28894151 (86.25%) | 4606321 (13.75%) | 27341316 (94.63%) |
| T2-3 | 45786676 | 39427362 (86.11%) | 2174576 (5.52%) | 37252786 (94.48%) | 37252786 | 30769840 (82.60%) | 6482946 (17.40%) | 29296477 (95.21%) |

Note: Total Mapped: The total number of sequences mapped in the reference genome, and the percentage is Total Mapped/Clean Reads; Multiple Mapped: The total number of sequences aligned to multiple positions, and the percentage is Multiple Mapped/Total Mapped; Uniquely Mapped: The total number of sequences aligned to single position, and the percentage is Uniquely Mapped/Total Mapped; Map Events: The total number of mapping events; Mapped reads: The total number of Reads aligned to the gene region and the percentage is Mapped reads/Map Events; Mapped to Inter: The total number of Reads aligned to the intergenic region and the percentage is Mapped to Inter/Map Events; Mapped to exon: The total number of Reads aligned to the exon region and the percentage is Mapped to exon/Mapped reads.
