# Supplementary material for: Oxygenation alleviates waterlogging-caused damages to cherry rootstocks
Source: Mol Hortic. 2023 Apr 17;3:8. doi: 10.1186/s43897-023-00056-1 (PMC10515082; doi:10.1186/s43897-023-00056-1)
Supplement: Supplementary file 2 — Additional file 2: Table S1. Sequence of primers used for quantitative reverse-transcription PCR. Table S2. Summary of the sequence data analysis. Table S3. Summary of RNA-Seq map. Table S4. KEGG pathway enrichment of differentially expressed genes in T1 vs. CK comparison. Table S5. KEGG pathway enrichment of differentially expressed genes in T2 vs. CK comparison. Table S6. KEGG pathway enrichment of differentially expressed genes in T2 vs. T1 comparison. Table S7. Expression profiles of differentially expressed genes associated with energy production. Table S8. Expression profiles of differentially expressed genes in the ethylene metabolic pathway. Table S9. Expression profiles of differentially expressed genes in the abscisic acid metabolic pathway. Table S10. Expression profiles of differentially expressed genes in the cytokinin metabolic pathway. Table S11. Expression profiles of differentially expressed genes in the auxin metabolic pathway. Table S12. Expression profiles of differentially expressed genes in the gibberellin metabolic pathway. Table S13. Expression profiles of differentially expressed genes in the salicylic acid metabolic pathway. Table S14. Expression profiles of differentially expressed genes in the brassinosteroid metabolic pathway. Table S15. Expression profiles of differentially expressed genes related to stress-associated transcription factors. Table S16. Expression profiles of differentially expressed genes related to stress. [file 43897_2023_56_MOESM2_ESM.zip › Table S1-S16/Table S4.docx]

**Table S4 KEGG pathway enrichment of T1 vs CK.**

| **PathwayID** | **Pathway** | **level1** | **level2** | **Up_**  **number** | **Down_**  **number** | **DEG_**  **number** | **total_**  **number** | **Pvalue** |
| --- | --- | --- | --- | --- | --- | --- | --- | --- |
| pavi00196 | Photosynthesis - antenna proteins | Metabolism | Energy metabolism | 0 | 9 | 9 | 12 | 2.24E-09 |
| pavi00195 | Photosynthesis | Metabolism | Energy metabolism | 0 | 13 | 13 | 36 | 9.56E-08 |
| pavi00480 | Glutathione metabolism | Metabolism | Metabolism of other amino acids | 16 | 1 | 17 | 82 | 7.37E-06 |
| pavi00250 | Alanine, aspartate and glutamate metabolism | Metabolism | Amino acid metabolism | 5 | 5 | 10 | 46 | 0.000397873 |
| pavi00052 | Galactose metabolism | Metabolism | Carbohydrate metabolism | 8 | 1 | 9 | 40 | 0.000594817 |
| pavi00860 | Porphyrin metabolism | Metabolism | Metabolism of cofactors and vitamins | 2 | 7 | 9 | 41 | 0.000721556 |
| pavi00900 | Terpenoid backbone biosynthesis | Metabolism | Metabolism of terpenoids and polyketides | 5 | 4 | 9 | 42 | 0.000869505 |
| pavi00906 | Carotenoid biosynthesis | Metabolism | Metabolism of terpenoids and polyketides | 1 | 7 | 8 | 34 | 0.000876059 |
| pavi00940 | Phenylpropanoid biosynthesis | Metabolism | Biosynthesis of other secondary metabolites | 10 | 7 | 17 | 129 | 0.002288562 |
| pavi00630 | Glyoxylate and dicarboxylate metabolism | Metabolism | Carbohydrate metabolism | 1 | 8 | 9 | 52 | 0.004161147 |
| pavi04016 | MAPK signaling pathway - plant | Environmental Information Processing | Signal transduction | 11 | 2 | 13 | 103 | 0.010533304 |
| pavi00260 | Glycine, serine and threonine metabolism | Metabolism | Amino acid metabolism | 3 | 5 | 8 | 58 | 0.025520599 |
| pavi00910 | Nitrogen metabolism | Metabolism | Energy metabolism | 1 | 4 | 5 | 29 | 0.03102589 |
| pavi00997 | Biosynthesis of various secondary metabolites - part 3 | Metabolism | Biosynthesis of other secondary metabolites | 2 | 0 | 2 | 5 | 0.033987682 |
| pavi00904 | Diterpenoid biosynthesis | Metabolism | Metabolism of terpenoids and polyketides | 3 | 1 | 4 | 22 | 0.043987471 |
| pavi00410 | beta-Alanine metabolism | Metabolism | Metabolism of other amino acids | 4 | 1 | 5 | 33 | 0.050763408 |
| pavi00270 | Cysteine and methionine metabolism | Metabolism | Amino acid metabolism | 8 | 1 | 9 | 79 | 0.054514277 |
| pavi00220 | Arginine biosynthesis | Metabolism | Amino acid metabolism | 1 | 4 | 5 | 34 | 0.056625975 |
| pavi00942 | Anthocyanin biosynthesis | Metabolism | Biosynthesis of other secondary metabolites | 1 | 0 | 1 | 1 | 0.062195782 |
| pavi00908 | Zeatin biosynthesis | Metabolism | Metabolism of terpenoids and polyketides | 1 | 2 | 3 | 17 | 0.084658811 |
| pavi00030 | Pentose phosphate pathway | Metabolism | Carbohydrate metabolism | 3 | 2 | 5 | 39 | 0.091492941 |
| pavi00905 | Brassinosteroid biosynthesis | Metabolism | Metabolism of terpenoids and polyketides | 3 | 0 | 3 | 18 | 0.097122553 |
| pavi00520 | Amino sugar and nucleotide sugar metabolism | Metabolism | Carbohydrate metabolism | 9 | 1 | 10 | 105 | 0.115174246 |
| pavi04626 | Plant-pathogen interaction | Organismal Systems | Environmental adaptation | 9 | 4 | 13 | 147 | 0.12332606 |
| pavi00710 | Carbon fixation in photosynthetic organisms | Metabolism | Energy metabolism | 1 | 5 | 6 | 55 | 0.123957512 |
| pavi04075 | Plant hormone signal transduction | Environmental Information Processing | Signal transduction | 10 | 8 | 18 | 222 | 0.145369912 |
| pavi00591 | Linoleic acid metabolism | Metabolism | Lipid metabolism | 1 | 1 | 2 | 11 | 0.14638589 |
| pavi00430 | Taurine and hypotaurine metabolism | Metabolism | Metabolism of other amino acids | 2 | 0 | 2 | 12 | 0.168786645 |
| pavi00280 | Valine, leucine and isoleucine degradation | Metabolism | Amino acid metabolism | 4 | 0 | 4 | 35 | 0.169971222 |
| pavi00945 | Stilbenoid, diarylheptanoid and gingerol biosynthesis | Metabolism | Biosynthesis of other secondary metabolites | 2 | 1 | 3 | 24 | 0.184593018 |
| pavi00300 | Lysine biosynthesis | Metabolism | Amino acid metabolism | 2 | 0 | 2 | 14 | 0.215011661 |
| pavi00531 | Glycosaminoglycan degradation | Metabolism | Glycan biosynthesis and metabolism | 2 | 0 | 2 | 14 | 0.215011661 |
| pavi00604 | Glycosphingolipid biosynthesis - ganglio series | Metabolism | Glycan biosynthesis and metabolism | 1 | 0 | 1 | 4 | 0.226603922 |
| pavi00053 | Ascorbate and aldarate metabolism | Metabolism | Carbohydrate metabolism | 4 | 0 | 4 | 40 | 0.235491435 |
| pavi00650 | Butanoate metabolism | Metabolism | Carbohydrate metabolism | 2 | 0 | 2 | 15 | 0.238550426 |
| pavi00740 | Riboflavin metabolism | Metabolism | Metabolism of cofactors and vitamins | 0 | 2 | 2 | 15 | 0.238550426 |
| pavi04146 | Peroxisome | Cellular Processes | Transport and catabolism | 3 | 3 | 6 | 70 | 0.267531775 |
| pavi00903 | Limonene and pinene degradation | Metabolism | Metabolism of terpenoids and polyketides | 1 | 0 | 1 | 5 | 0.274757983 |
| pavi00592 | alpha-Linolenic acid metabolism | Metabolism | Lipid metabolism | 2 | 1 | 3 | 31 | 0.30297245 |
| pavi02010 | ABC transporters | Environmental Information Processing | Membrane transport | 1 | 1 | 2 | 19 | 0.332912754 |
| pavi04070 | Phosphatidylinositol signaling system | Environmental Information Processing | Signal transduction | 4 | 0 | 4 | 47 | 0.334719197 |
| pavi00130 | Ubiquinone and other terpenoid-quinone biosynthesis | Metabolism | Metabolism of cofactors and vitamins | 0 | 3 | 3 | 34 | 0.355312284 |
| pavi00603 | Glycosphingolipid biosynthesis - globo and isoglobo series | Metabolism | Glycan biosynthesis and metabolism | 1 | 0 | 1 | 8 | 0.402030454 |
| pavi00010 | Glycolysis / Gluconeogenesis | Metabolism | Carbohydrate metabolism | 4 | 2 | 6 | 82 | 0.40217372 |
| pavi00400 | Phenylalanine, tyrosine and tryptophan biosynthesis | Metabolism | Amino acid metabolism | 2 | 1 | 3 | 38 | 0.424168466 |
| pavi00100 | Steroid biosynthesis | Metabolism | Lipid metabolism | 0 | 2 | 2 | 26 | 0.487580701 |
| pavi00941 | Flavonoid biosynthesis | Metabolism | Biosynthesis of other secondary metabolites | 2 | 0 | 2 | 27 | 0.507860809 |
| pavi00750 | Vitamin B6 metabolism | Metabolism | Metabolism of cofactors and vitamins | 0 | 1 | 1 | 12 | 0.537798847 |
| pavi00920 | Sulfur metabolism | Metabolism | Energy metabolism | 0 | 2 | 2 | 29 | 0.54682402 |
| pavi04712 | Circadian rhythm - plant | Organismal Systems | Environmental adaptation | 0 | 2 | 2 | 29 | 0.54682402 |
| pavi00051 | Fructose and mannose metabolism | Metabolism | Carbohydrate metabolism | 1 | 2 | 3 | 46 | 0.552655 |
| pavi00562 | Inositol phosphate metabolism | Metabolism | Carbohydrate metabolism | 3 | 0 | 3 | 46 | 0.552655 |
| pavi00071 | Fatty acid degradation | Metabolism | Lipid metabolism | 2 | 0 | 2 | 30 | 0.56548478 |
| pavi00770 | Pantothenate and CoA biosynthesis | Metabolism | Metabolism of cofactors and vitamins | 2 | 0 | 2 | 30 | 0.56548478 |
| pavi00511 | Other glycan degradation | Metabolism | Glycan biosynthesis and metabolism | 1 | 0 | 1 | 13 | 0.566639397 |
| pavi00966 | Glucosinolate biosynthesis | Metabolism | Biosynthesis of other secondary metabolites | 1 | 0 | 1 | 13 | 0.566639397 |
| pavi00909 | Sesquiterpenoid and triterpenoid biosynthesis | Metabolism | Metabolism of terpenoids and polyketides | 0 | 1 | 1 | 14 | 0.593687684 |
| pavi00450 | Selenocompound metabolism | Metabolism | Metabolism of other amino acids | 1 | 0 | 1 | 15 | 0.619054631 |
| pavi00330 | Arginine and proline metabolism | Metabolism | Amino acid metabolism | 2 | 1 | 3 | 51 | 0.623840319 |
| pavi00460 | Cyanoamino acid metabolism | Metabolism | Metabolism of other amino acids | 1 | 2 | 3 | 51 | 0.623840319 |
| pavi00340 | Histidine metabolism | Metabolism | Amino acid metabolism | 1 | 0 | 1 | 16 | 0.642844323 |
| pavi00563 | Glycosylphosphatidylinositol (GPI)-anchor biosynthesis | Metabolism | Glycan biosynthesis and metabolism | 1 | 0 | 1 | 16 | 0.642844323 |
| pavi00590 | Arachidonic acid metabolism | Metabolism | Lipid metabolism | 1 | 0 | 1 | 16 | 0.642844323 |
| pavi00290 | Valine, leucine and isoleucine biosynthesis | Metabolism | Amino acid metabolism | 1 | 0 | 1 | 17 | 0.665154427 |
| pavi00780 | Biotin metabolism | Metabolism | Metabolism of cofactors and vitamins | 1 | 0 | 1 | 17 | 0.665154427 |
| pavi00730 | Thiamine metabolism | Metabolism | Metabolism of cofactors and vitamins | 0 | 1 | 1 | 18 | 0.68607659 |
| pavi00670 | One carbon pool by folate | Metabolism | Metabolism of cofactors and vitamins | 0 | 1 | 1 | 19 | 0.705696803 |
| pavi00760 | Nicotinate and nicotinamide metabolism | Metabolism | Metabolism of cofactors and vitamins | 0 | 1 | 1 | 19 | 0.705696803 |
| pavi00620 | Pyruvate metabolism | Metabolism | Carbohydrate metabolism | 3 | 1 | 4 | 77 | 0.715683527 |
| pavi00565 | Ether lipid metabolism | Metabolism | Lipid metabolism | 1 | 0 | 1 | 21 | 0.741349145 |
| pavi00230 | Purine metabolism | Metabolism | Nucleotide metabolism | 1 | 2 | 3 | 64 | 0.770902209 |
| pavi00360 | Phenylalanine metabolism | Metabolism | Amino acid metabolism | 1 | 0 | 1 | 24 | 0.786924643 |
| pavi00240 | Pyrimidine metabolism | Metabolism | Nucleotide metabolism | 0 | 2 | 2 | 46 | 0.790663837 |
| pavi00062 | Fatty acid elongation | Metabolism | Lipid metabolism | 0 | 1 | 1 | 25 | 0.800263601 |
| pavi00310 | Lysine degradation | Metabolism | Amino acid metabolism | 1 | 0 | 1 | 26 | 0.812770917 |
| pavi00500 | Starch and sucrose metabolism | Metabolism | Carbohydrate metabolism | 3 | 2 | 5 | 113 | 0.842380903 |
| pavi00640 | Propanoate metabolism | Metabolism | Carbohydrate metabolism | 1 | 0 | 1 | 30 | 0.85546979 |
| pavi00513 | Various types of N-glycan biosynthesis | Metabolism | Glycan biosynthesis and metabolism | 1 | 0 | 1 | 32 | 0.873029216 |
| pavi04130 | SNARE interactions in vesicular transport | Genetic Information Processing | Folding, sorting and degradation | 1 | 0 | 1 | 34 | 0.888463437 |
| pavi00380 | Tryptophan metabolism | Metabolism | Amino acid metabolism | 1 | 0 | 1 | 35 | 0.895464913 |
| pavi00350 | Tyrosine metabolism | Metabolism | Amino acid metabolism | 1 | 0 | 1 | 38 | 0.913950392 |
| pavi00020 | Citrate cycle (TCA cycle) | Metabolism | Carbohydrate metabolism | 0 | 1 | 1 | 40 | 0.92442694 |
| pavi00040 | Pentose and glucuronate interconversions | Metabolism | Carbohydrate metabolism | 2 | 0 | 2 | 68 | 0.931806573 |
| pavi04141 | Protein processing in endoplasmic reticulum | Genetic Information Processing | Folding, sorting and degradation | 5 | 2 | 7 | 178 | 0.935244302 |
| pavi00564 | Glycerophospholipid metabolism | Metabolism | Lipid metabolism | 1 | 1 | 2 | 73 | 0.947817793 |
| pavi03018 | RNA degradation | Genetic Information Processing | Folding, sorting and degradation | 2 | 0 | 2 | 79 | 0.962333294 |
| pavi03420 | Nucleotide excision repair | Genetic Information Processing | Replication and repair | 0 | 1 | 1 | 51 | 0.963043039 |
| pavi00561 | Glycerolipid metabolism | Metabolism | Lipid metabolism | 1 | 0 | 1 | 53 | 0.967558071 |
| pavi04145 | Phagosome | Cellular Processes | Transport and catabolism | 1 | 0 | 1 | 57 | 0.975006263 |
| pavi03008 | Ribosome biogenesis in eukaryotes | Genetic Information Processing | Translation | 1 | 0 | 1 | 74 | 0.991778111 |
| pavi00190 | Oxidative phosphorylation | Metabolism | Energy metabolism | 0 | 1 | 1 | 86 | 0.99626132 |
| pavi04144 | Endocytosis | Cellular Processes | Transport and catabolism | 2 | 0 | 2 | 130 | 0.997978436 |
| pavi04120 | Ubiquitin mediated proteolysis | Genetic Information Processing | Folding, sorting and degradation | 0 | 1 | 1 | 112 | 0.999328385 |
| pavi03040 | Spliceosome | Genetic Information Processing | Transcription | 2 | 0 | 2 | 166 | 0.999771171 |
| pavi03010 | Ribosome | Genetic Information Processing | Translation | 1 | 0 | 1 | 239 | 0.999999873 |
