# Supplementary material for: Oxygenation alleviates waterlogging-caused damages to cherry rootstocks
Source: Mol Hortic. 2023 Apr 17;3:8. doi: 10.1186/s43897-023-00056-1 (PMC10515082; doi:10.1186/s43897-023-00056-1)
Supplement: Supplementary file 2 — Additional file 2: Table S1. Sequence of primers used for quantitative reverse-transcription PCR. Table S2. Summary of the sequence data analysis. Table S3. Summary of RNA-Seq map. Table S4. KEGG pathway enrichment of differentially expressed genes in T1 vs. CK comparison. Table S5. KEGG pathway enrichment of differentially expressed genes in T2 vs. CK comparison. Table S6. KEGG pathway enrichment of differentially expressed genes in T2 vs. T1 comparison. Table S7. Expression profiles of differentially expressed genes associated with energy production. Table S8. Expression profiles of differentially expressed genes in the ethylene metabolic pathway. Table S9. Expression profiles of differentially expressed genes in the abscisic acid metabolic pathway. Table S10. Expression profiles of differentially expressed genes in the cytokinin metabolic pathway. Table S11. Expression profiles of differentially expressed genes in the auxin metabolic pathway. Table S12. Expression profiles of differentially expressed genes in the gibberellin metabolic pathway. Table S13. Expression profiles of differentially expressed genes in the salicylic acid metabolic pathway. Table S14. Expression profiles of differentially expressed genes in the brassinosteroid metabolic pathway. Table S15. Expression profiles of differentially expressed genes related to stress-associated transcription factors. Table S16. Expression profiles of differentially expressed genes related to stress. [file 43897_2023_56_MOESM2_ESM.zip › Table S1-S16/Table S5.docx]

**Table S5 KEGG pathway enrichment of T2 vs CK.**

| **PathwayID** | **Pathway** | **level1** | **level2** | **Up_**  **number** | **Down_**  **number** | **DEG_**  **number** | **total_**  **number** | **Pvalue** |
| --- | --- | --- | --- | --- | --- | --- | --- | --- |
| pavi00480 | Glutathione metabolism | Metabolism | Metabolism of other amino acids | 10 | 0 | 10 | 82 | 4.73E-05 |
| pavi00052 | Galactose metabolism | Metabolism | Carbohydrate metabolism | 7 | 0 | 7 | 40 | 6.74E-05 |
| pavi04016 | MAPK signaling pathway - plant | Environmental Information Processing | Signal transduction | 9 | 1 | 10 | 103 | 0.000328892 |
| pavi00196 | Photosynthesis - antenna proteins | Metabolism | Energy metabolism | 0 | 3 | 3 | 12 | 0.003337177 |
| pavi00997 | Biosynthesis of various secondary metabolites - part 3 | Metabolism | Biosynthesis of other secondary metabolites | 2 | 0 | 2 | 5 | 0.006598863 |
| pavi00250 | Alanine, aspartate and glutamate metabolism | Metabolism | Amino acid metabolism | 2 | 2 | 4 | 46 | 0.03234778 |
| pavi00040 | Pentose and glucuronate interconversions | Metabolism | Carbohydrate metabolism | 3 | 2 | 5 | 68 | 0.032993802 |
| pavi04626 | Plant-pathogen interaction | Organismal Systems | Environmental adaptation | 3 | 5 | 8 | 147 | 0.039539206 |
| pavi00940 | Phenylpropanoid biosynthesis | Metabolism | Biosynthesis of other secondary metabolites | 5 | 2 | 7 | 129 | 0.053410365 |
| pavi00740 | Riboflavin metabolism | Metabolism | Metabolism of cofactors and vitamins | 0 | 2 | 2 | 15 | 0.05834002 |
| pavi04075 | Plant hormone signal transduction | Environmental Information Processing | Signal transduction | 7 | 3 | 10 | 222 | 0.067231345 |
| pavi00908 | Zeatin biosynthesis | Metabolism | Metabolism of terpenoids and polyketides | 1 | 1 | 2 | 17 | 0.073041495 |
| pavi00053 | Ascorbate and aldarate metabolism | Metabolism | Carbohydrate metabolism | 3 | 0 | 3 | 40 | 0.088165033 |
| pavi00562 | Inositol phosphate metabolism | Metabolism | Carbohydrate metabolism | 3 | 0 | 3 | 46 | 0.121218001 |
| pavi04070 | Phosphatidylinositol signaling system | Environmental Information Processing | Signal transduction | 3 | 0 | 3 | 47 | 0.127119712 |
| pavi00520 | Amino sugar and nucleotide sugar metabolism | Metabolism | Carbohydrate metabolism | 5 | 0 | 5 | 105 | 0.144276104 |
| pavi00460 | Cyanoamino acid metabolism | Metabolism | Metabolism of other amino acids | 1 | 2 | 3 | 51 | 0.151706261 |
| pavi00270 | Cysteine and methionine metabolism | Metabolism | Amino acid metabolism | 4 | 0 | 4 | 79 | 0.155900354 |
| pavi00710 | Carbon fixation in photosynthetic organisms | Metabolism | Energy metabolism | 1 | 2 | 3 | 55 | 0.177671263 |
| pavi00910 | Nitrogen metabolism | Metabolism | Energy metabolism | 0 | 2 | 2 | 29 | 0.17842928 |
| pavi00410 | beta-Alanine metabolism | Metabolism | Metabolism of other amino acids | 1 | 1 | 2 | 33 | 0.217312712 |
| pavi00906 | Carotenoid biosynthesis | Metabolism | Metabolism of terpenoids and polyketides | 0 | 2 | 2 | 34 | 0.22716165 |
| pavi00400 | Phenylalanine, tyrosine and tryptophan biosynthesis | Metabolism | Amino acid metabolism | 1 | 1 | 2 | 38 | 0.266799902 |
| pavi00430 | Taurine and hypotaurine metabolism | Metabolism | Metabolism of other amino acids | 1 | 0 | 1 | 12 | 0.275871432 |
| pavi00860 | Porphyrin metabolism | Metabolism | Metabolism of cofactors and vitamins | 2 | 0 | 2 | 41 | 0.296566802 |
| pavi00900 | Terpenoid backbone biosynthesis | Metabolism | Metabolism of terpenoids and polyketides | 0 | 2 | 2 | 42 | 0.306459733 |
| pavi00909 | Sesquiterpenoid and triterpenoid biosynthesis | Metabolism | Metabolism of terpenoids and polyketides | 0 | 1 | 1 | 14 | 0.313869588 |
| pavi00650 | Butanoate metabolism | Metabolism | Carbohydrate metabolism | 1 | 0 | 1 | 15 | 0.3321217 |
| pavi00500 | Starch and sucrose metabolism | Metabolism | Carbohydrate metabolism | 2 | 2 | 4 | 113 | 0.35139597 |
| pavi00905 | Brassinosteroid biosynthesis | Metabolism | Metabolism of terpenoids and polyketides | 1 | 0 | 1 | 18 | 0.384043586 |
| pavi00330 | Arginine and proline metabolism | Metabolism | Amino acid metabolism | 1 | 1 | 2 | 51 | 0.393770197 |
| pavi00760 | Nicotinate and nicotinamide metabolism | Metabolism | Metabolism of cofactors and vitamins | 0 | 1 | 1 | 19 | 0.400446773 |
| pavi00904 | Diterpenoid biosynthesis | Metabolism | Metabolism of terpenoids and polyketides | 0 | 1 | 1 | 22 | 0.447106291 |
| pavi00945 | Stilbenoid, diarylheptanoid and gingerol biosynthesis | Metabolism | Biosynthesis of other secondary metabolites | 1 | 0 | 1 | 24 | 0.476196883 |
| pavi00062 | Fatty acid elongation | Metabolism | Lipid metabolism | 0 | 1 | 1 | 25 | 0.490168768 |
| pavi00941 | Flavonoid biosynthesis | Metabolism | Biosynthesis of other secondary metabolites | 1 | 0 | 1 | 27 | 0.517015237 |
| pavi04712 | Circadian rhythm - plant | Organismal Systems | Environmental adaptation | 0 | 1 | 1 | 29 | 0.542461707 |
| pavi00220 | Arginine biosynthesis | Metabolism | Amino acid metabolism | 0 | 1 | 1 | 34 | 0.600418265 |
| pavi00010 | Glycolysis / Gluconeogenesis | Metabolism | Carbohydrate metabolism | 0 | 2 | 2 | 82 | 0.645928171 |
| pavi00030 | Pentose phosphate pathway | Metabolism | Carbohydrate metabolism | 0 | 1 | 1 | 39 | 0.651098853 |
| pavi00051 | Fructose and mannose metabolism | Metabolism | Carbohydrate metabolism | 0 | 1 | 1 | 46 | 0.711529991 |
| pavi00240 | Pyrimidine metabolism | Metabolism | Nucleotide metabolism | 0 | 1 | 1 | 46 | 0.711529991 |
| pavi00630 | Glyoxylate and dicarboxylate metabolism | Metabolism | Carbohydrate metabolism | 1 | 0 | 1 | 52 | 0.754997025 |
| pavi00970 | Aminoacyl-tRNA biosynthesis | Genetic Information Processing | Translation | 1 | 0 | 1 | 53 | 0.761582404 |
| pavi04145 | Phagosome | Cellular Processes | Transport and catabolism | 1 | 0 | 1 | 57 | 0.786217002 |
| pavi00260 | Glycine, serine and threonine metabolism | Metabolism | Amino acid metabolism | 0 | 1 | 1 | 58 | 0.791971117 |
| pavi00230 | Purine metabolism | Metabolism | Nucleotide metabolism | 1 | 0 | 1 | 64 | 0.823413658 |
| pavi04146 | Peroxisome | Cellular Processes | Transport and catabolism | 1 | 0 | 1 | 70 | 0.850144952 |
| pavi00564 | Glycerophospholipid metabolism | Metabolism | Lipid metabolism | 1 | 0 | 1 | 73 | 0.86196676 |
| pavi04144 | Endocytosis | Cellular Processes | Transport and catabolism | 2 | 0 | 2 | 130 | 0.866486884 |
| pavi00620 | Pyruvate metabolism | Metabolism | Carbohydrate metabolism | 0 | 1 | 1 | 77 | 0.876304617 |
| pavi03040 | Spliceosome | Genetic Information Processing | Transcription | 1 | 1 | 2 | 166 | 0.940136703 |
| pavi04141 | Protein processing in endoplasmic reticulum | Genetic Information Processing | Folding, sorting and degradation | 1 | 0 | 1 | 178 | 0.992558199 |
