# Supplementary material for: Oxygenation alleviates waterlogging-caused damages to cherry rootstocks
Source: Mol Hortic. 2023 Apr 17;3:8. doi: 10.1186/s43897-023-00056-1 (PMC10515082; doi:10.1186/s43897-023-00056-1)
Supplement: Supplementary file 2 — Additional file 2: Table S1. Sequence of primers used for quantitative reverse-transcription PCR. Table S2. Summary of the sequence data analysis. Table S3. Summary of RNA-Seq map. Table S4. KEGG pathway enrichment of differentially expressed genes in T1 vs. CK comparison. Table S5. KEGG pathway enrichment of differentially expressed genes in T2 vs. CK comparison. Table S6. KEGG pathway enrichment of differentially expressed genes in T2 vs. T1 comparison. Table S7. Expression profiles of differentially expressed genes associated with energy production. Table S8. Expression profiles of differentially expressed genes in the ethylene metabolic pathway. Table S9. Expression profiles of differentially expressed genes in the abscisic acid metabolic pathway. Table S10. Expression profiles of differentially expressed genes in the cytokinin metabolic pathway. Table S11. Expression profiles of differentially expressed genes in the auxin metabolic pathway. Table S12. Expression profiles of differentially expressed genes in the gibberellin metabolic pathway. Table S13. Expression profiles of differentially expressed genes in the salicylic acid metabolic pathway. Table S14. Expression profiles of differentially expressed genes in the brassinosteroid metabolic pathway. Table S15. Expression profiles of differentially expressed genes related to stress-associated transcription factors. Table S16. Expression profiles of differentially expressed genes related to stress. [file 43897_2023_56_MOESM2_ESM.zip › Table S1-S16/Table S6.docx]

**Table S6 KEGG pathway enrichment of T2 vs T1.**

| **PathwayID** | **Pathway** | **level1** | **level2** | **Up_**  **number** | **Down_**  **number** | **DEG_**  **number** | **total_**  **number** | **Pvalue** |
| --- | --- | --- | --- | --- | --- | --- | --- | --- |
| pavi00910 | Nitrogen metabolism | Metabolism | Energy metabolism | 3 | 1 | 4 | 29 | 0.000789471 |
| pavi00053 | Ascorbate and aldarate metabolism | Metabolism | Carbohydrate metabolism | 1 | 3 | 4 | 40 | 0.002691019 |
| pavi04626 | Plant-pathogen interaction | Organismal Systems | Environmental adaptation | 0 | 7 | 7 | 147 | 0.005557486 |
| pavi00909 | Sesquiterpenoid and triterpenoid biosynthesis | Metabolism | Metabolism of terpenoids and polyketides | 1 | 1 | 2 | 14 | 0.017627924 |
| pavi00908 | Zeatin biosynthesis | Metabolism | Metabolism of terpenoids and polyketides | 0 | 2 | 2 | 17 | 0.02560654 |
| pavi04075 | Plant hormone signal transduction | Environmental Information Processing | Signal transduction | 2 | 5 | 7 | 222 | 0.044321483 |
| pavi04016 | MAPK signaling pathway - plant | Environmental Information Processing | Signal transduction | 0 | 4 | 4 | 103 | 0.065585591 |
| pavi00520 | Amino sugar and nucleotide sugar metabolism | Metabolism | Carbohydrate metabolism | 2 | 2 | 4 | 105 | 0.069402445 |
| pavi00040 | Pentose and glucuronate interconversions | Metabolism | Carbohydrate metabolism | 1 | 2 | 3 | 68 | 0.079283616 |
| pavi00220 | Arginine biosynthesis | Metabolism | Amino acid metabolism | 2 | 0 | 2 | 34 | 0.090066673 |
| pavi00480 | Glutathione metabolism | Metabolism | Metabolism of other amino acids | 1 | 2 | 3 | 82 | 0.121513297 |
| pavi00860 | Porphyrin metabolism | Metabolism | Metabolism of cofactors and vitamins | 1 | 1 | 2 | 41 | 0.123396378 |
| pavi00940 | Phenylpropanoid biosynthesis | Metabolism | Biosynthesis of other secondary metabolites | 3 | 1 | 4 | 129 | 0.123685682 |
| pavi00562 | Inositol phosphate metabolism | Metabolism | Carbohydrate metabolism | 1 | 1 | 2 | 46 | 0.148761328 |
| pavi00330 | Arginine and proline metabolism | Metabolism | Amino acid metabolism | 2 | 0 | 2 | 51 | 0.175081748 |
| pavi00966 | Glucosinolate biosynthesis | Metabolism | Biosynthesis of other secondary metabolites | 0 | 1 | 1 | 13 | 0.177263386 |
| pavi00970 | Aminoacyl-tRNA biosynthesis | Genetic Information Processing | Translation | 2 | 0 | 2 | 53 | 0.185817331 |
| pavi00710 | Carbon fixation in photosynthetic organisms | Metabolism | Energy metabolism | 1 | 1 | 2 | 55 | 0.196647891 |
| pavi00290 | Valine, leucine and isoleucine biosynthesis | Metabolism | Amino acid metabolism | 0 | 1 | 1 | 17 | 0.225312345 |
| pavi01040 | Biosynthesis of unsaturated fatty acids | Metabolism | Lipid metabolism | 1 | 0 | 1 | 20 | 0.259530438 |
| pavi00904 | Diterpenoid biosynthesis | Metabolism | Metabolism of terpenoids and polyketides | 1 | 0 | 1 | 22 | 0.281513461 |
| pavi00945 | Stilbenoid, diarylheptanoid and gingerol biosynthesis | Metabolism | Biosynthesis of other secondary metabolites | 1 | 0 | 1 | 24 | 0.302855372 |
| pavi00062 | Fatty acid elongation | Metabolism | Lipid metabolism | 1 | 0 | 1 | 25 | 0.313291668 |
| pavi00100 | Steroid biosynthesis | Metabolism | Lipid metabolism | 1 | 0 | 1 | 26 | 0.323574532 |
| pavi00770 | Pantothenate and CoA biosynthesis | Metabolism | Metabolism of cofactors and vitamins | 0 | 1 | 1 | 30 | 0.363215479 |
| pavi00410 | beta-Alanine metabolism | Metabolism | Metabolism of other amino acids | 1 | 0 | 1 | 33 | 0.391440576 |
| pavi03410 | Base excision repair | Genetic Information Processing | Replication and repair | 1 | 0 | 1 | 33 | 0.391440576 |
| pavi00906 | Carotenoid biosynthesis | Metabolism | Metabolism of terpenoids and polyketides | 1 | 0 | 1 | 34 | 0.400573119 |
| pavi00280 | Valine, leucine and isoleucine degradation | Metabolism | Amino acid metabolism | 0 | 1 | 1 | 35 | 0.409571066 |
| pavi00400 | Phenylalanine, tyrosine and tryptophan biosynthesis | Metabolism | Amino acid metabolism | 0 | 1 | 1 | 38 | 0.435776672 |
| pavi00052 | Galactose metabolism | Metabolism | Carbohydrate metabolism | 0 | 1 | 1 | 40 | 0.452609073 |
| pavi00240 | Pyrimidine metabolism | Metabolism | Nucleotide metabolism | 1 | 0 | 1 | 46 | 0.500202881 |
| pavi00250 | Alanine, aspartate and glutamate metabolism | Metabolism | Amino acid metabolism | 1 | 0 | 1 | 46 | 0.500202881 |
| pavi00500 | Starch and sucrose metabolism | Metabolism | Carbohydrate metabolism | 1 | 1 | 2 | 113 | 0.505575439 |
| pavi04070 | Phosphatidylinositol signaling system | Environmental Information Processing | Signal transduction | 0 | 1 | 1 | 47 | 0.507729946 |
| pavi00460 | Cyanoamino acid metabolism | Metabolism | Metabolism of other amino acids | 1 | 0 | 1 | 51 | 0.536740969 |
| pavi00630 | Glyoxylate and dicarboxylate metabolism | Metabolism | Carbohydrate metabolism | 1 | 0 | 1 | 52 | 0.543727327 |
| pavi00620 | Pyruvate metabolism | Metabolism | Carbohydrate metabolism | 0 | 1 | 1 | 77 | 0.688367185 |
| pavi00270 | Cysteine and methionine metabolism | Metabolism | Amino acid metabolism | 0 | 1 | 1 | 79 | 0.697763466 |
| pavi04120 | Ubiquitin mediated proteolysis | Genetic Information Processing | Folding, sorting and degradation | 0 | 1 | 1 | 112 | 0.818078548 |
| pavi04144 | Endocytosis | Cellular Processes | Transport and catabolism | 1 | 0 | 1 | 130 | 0.862352565 |
